# Probabilistic transmission models incorporating sequencing data for healthcare-associated *Clostridioides difficile* outperform heuristic rules and identify strain-specific differences in transmission

**DOI:** 10.1371/journal.pcbi.1008417

**Published:** 2021-01-14

**Authors:** David W. Eyre, Mirjam Laager, A. Sarah Walker, Ben S. Cooper, Daniel J. Wilson, on behalf of the CDC Modeling Infectious Diseases in Healthcare Program (MInD-Healthcare)

**Affiliations:** 1 Big Data Institute, Nuffield Department of Population Health, University of Oxford, United Kingdom; 2 Nuffield Department of Medicine, University of Oxford, United Kingdom; 3 Health Protection Research Unit in Healthcare Associated Infections and Antimicrobial Resistance, University of Oxford, United Kingdom; University of Zurich, SWITZERLAND

## Abstract

Fitting stochastic transmission models to electronic patient data can offer detailed insights into the transmission of healthcare-associated infections and improve infection control. Pathogen whole-genome sequencing may improve the precision of model inferences, but computational constraints have limited modelling applications predominantly to small datasets and specific outbreaks, whereas large-scale sequencing studies have mostly relied on simple rules for identifying/excluding plausible transmission. We present a novel approach for integrating detailed epidemiological data on patient contact networks in hospitals with large-scale pathogen sequencing data. We apply our approach to study *Clostridioides difficile* transmission using a dataset of 1223 infections in Oxfordshire, UK, 2007–2011. 262 (21% [95% credibility interval 20–22%]) infections were estimated to have been acquired from another known case. There was heterogeneity by sequence type (ST) in the proportion of cases acquired from another case with the highest rates in ST1 (ribotype-027), ST42 (ribotype-106) and ST3 (ribotype-001). These same STs also had higher rates of transmission mediated via environmental contamination/spores persisting after patient discharge/recovery; for ST1 these persisted longer than for most other STs except ST3 and ST42. We also identified variation in transmission between hospitals, medical specialties and over time; by 2011 nearly all transmission from known cases had ceased in our hospitals. Our findings support previous work suggesting only a minority of *C*. *difficile* infections are acquired from known cases but highlight a greater role for environmental contamination than previously thought. Our approach is applicable to other healthcare-associated infections. Our findings have important implications for effective control of *C*. *difficile*.

## Author summary

Preventing infections spreading in hospitals is a major priority for healthcare systems globally. Mathematical models can be used with electronic hospital records to reconstruct how infections spread, which in turn can help guide infection control interventions. Sequencing the genetic code (DNA) of the bacteria that cause infections can help to follow when transmission is occurring, as bacterial DNA from two patients infected from the same source will likely be very similar. Our paper describes a new statistical method for combining hospital records and sequencing data to track infections. We use our approach to study over 1200 infections of *Clostridioides difficile* (C. diff.), which is a common cause of diarrhoea in hospitals. We show that only a minority of infections are acquired from other unwell patients, but the amount of spread varies by the subtype of C. diff involved. We also show that different C. diff subtypes survive in the hospital environment for longer than others and may need enhanced control strategies. We also can detect differences in spread at different hospitals and show that by the end of the study we had largely eliminated transmission of C. diff from unwell patients in our hospitals.

## Introduction

Healthcare-associated infections have a substantial impact on the health and well-being of hospitalised patients. Many are preventable through effective infection prevention and control. However how to best target these interventions crucially depends on understanding how infection is transmitted.

Using data collected as part of routine healthcare, e.g. microbiology results and patient admission data, stochastic transmission models can be used to estimate important transmission properties, in a way that is robust to the noisy and incomplete data available, and also provides an indication of the uncertainty surrounding estimates made [1]. For example, the parameters governing transmission can be explored and the relative importance of different routes of transmission, the properties of different pathogen strains, and patient and hospital-level risk factors for transmission estimated. Although the relative extent of transmission on particular hospital wards or from specific patient groups can be monitored via incidence data alone, use of these models allows these differences to be estimated with much greater accuracy, particularly in endemic diseases where there are often multiple potentially plausible sources for each infection. As such, these models can also be used to provide metrics of infection control performance within and between healthcare institutions.

The availability of pathogen whole-genome sequencing data has enabled greater precision around estimates of who infected whom, e.g. [2–4], and therefore also of the underlying transmission parameters. These data can be used in a relatively simple way to exclude large numbers of potential transmission sources if these infections differ substantially from a given case. However, approaches that set a threshold for excluding transmission may falsely exclude some transmission events and also may still leave multiple plausible sources of infection all related within the threshold chosen. In contrast, probabilistic approaches can avoid hard cut-offs and can also represent which of several potential sources is most likely based on genomic and epidemiological data.

Several approaches have been adopted for integrating pathogen genomic and epidemiological data to study transmission. Some attempt to jointly reconstruct the transmission events (the transmission tree) and the phylogenetic relationships between the resulting sequences e.g. [5,6]. This has the advantage of letting the phylogenetic data inform the transmission tree and, vice versa, the epidemiological data inform the genomic analysis. However, these approaches are computationally intensive, and so have been typically applied to smaller datasets with relatively sparse epidemiological data and not, for example, representing the complex contact network between patients admitted to different hospital wards over time. The alternative approach is to first fix the phylogenetic relationship between sequences and then to fit an epidemiological model contingent on this or a distance-based summary of the fixed phylogeny. This approach has been implemented by several authors, e.g. to study community-based transmission of tuberculosis [7] and healthcare-associated transmission of methicillin-resistant *Staphylococcus aureus* [4]. However, much of the literature is focused on outbreak investigation rather than on understanding the transmission of an endemic disease.

Here we describe a novel approach for integrating pathogen genomic data into a transmission model. We build on an approach we have used in previous descriptive studies[2,8,9] for interpreting genetic distances between sequenced isolates based on coalescent theory using rates of mutation and within host diversity. We also develop a sophisticated transmission model for healthcare-associated *Clostridioides difficile* (formerly *Clostridium difficile*) and use this to study endemic transmission within Oxfordshire, UK over 3.5 years, analysing data from a previous descriptive study [2].

*C*. *difficile* is a major cause of healthcare-associated diarrhoea and can cause severe and life-threatening infections. *C*. *difficile* is shed in the faeces of infected patients, including as hardy spores which can persist in the environment for days to months. Within hospitals *C*. *difficile* can be spread between patients via the hands of healthcare workers, shared equipment and the wider hospital environment. Patients acquire *C*. *difficile* by ingestion. The onset of symptoms frequently occurs following antibiotic exposure, which disrupts the normal faecal microbiome, and allows *C*. *difficile* to flourish and cause toxin-mediated inflammation of the gut.

Over the last ten years substantial reductions in *C*. *difficile* incidence have been seen in the UK, with varying explanations for this postulated, including reduced use of fluroquinolone antibiotics [10], but also potentially better infection control [11]. Whilst others have applied probabilistic transmission modelling approaches to study *C*. *difficile* [12–14], this has not been done previously using genomic data and representations of the disease process vary. For example, using a subset of the same data from Oxfordshire that we study here, one study used only more limited typing data rather than whole-genome sequencing to estimate the relative contribution of routes of transmission and differences in transmission rates between strains [15].

By fitting our transmission model to a large-scale dataset of over 1200 *C*. *difficile* infections and accompanying sequence data, we demonstrate significant heterogeneity between the transmission of different strains of *C*. *difficile* and show that both enhanced transmission rates from infected cases and enhanced environmental persistence may underlie the success of healthcare-associated strains. We are also able to highlight differences in transmission rates between hospitals, medical specialties and over time.

## Methods

### Ethics statement

De-identified patient data were obtained from the Infections in Oxfordshire Research Database (IORD) which has generic Research Ethics Committee, Health Research Authority and Confidentiality Advisory Group approvals (19/SC/0403, 19/CAG/0144) as a de-identified electronic research database.

We describe an approach for combining data from electronic patient records on hospital ward movements and whole-genome sequencing data from sampled cases to investigate transmission of healthcare-associated infection. We reconstruct the timing and source of infection events, which we denote the transmission tree, and determine key parameters which underlie the transmission process. Hospital admission and discharge times and the specific ward stays within each admission are assumed to be available for all patients (cases and non-cases). For patients diagnosed with an infection, we assume the time the diagnostic sample was obtained is known and a whole-genome sequence of the resulting isolate is available. We apply our methods to investigate the transmission of *C*. *difficile*, but applications to other healthcare-associated infections would also be possible.

### Transmission model

**Fig 1:**
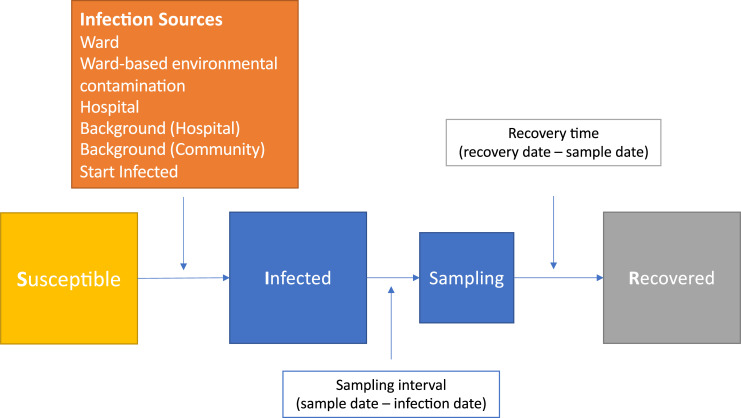
Transmission model states. Both boxes shaded blue represent an infectious patient.

Transmission is represented by an SIR (susceptible, infectious, recovered) epidemiological model, using discrete time in whole days (Fig 1). The use of discrete time reflects the precision of available data on ward admission and discharge times and patient sampling times. For the purposes of the model, once infected, patients were assumed to recover and then be protected from a repeat infection with the same sequence type, as only 41/1223 (3%) of patients in a previous study using the same dataset had evidence of the same infection persisting or recurring beyond 90 days, and many of these can be allowed for by modelling recovery with a sufficiently long-tailed distribution [2]. Patients with several genetically distinct infections are included in the dataset once for each infection with a different multi-locus sequence type (MLST). This duplication of patients had minimal impact on the numbers of patients overall, as the number of non-cases was considerably greater than the number of cases, there were 1223 cases overall and 291,669 non-cases, which is broadly similar to estimates of around 2 cases to 1000 non-cases in previous studies in other settings [16].

Patients can start the study period susceptible or infected (with probability α). We assume transmission occurs as a density dependent process, i.e. proportional to the number of infectious individuals present. (As ward occupancy in our setting is near 100%, this is very similar to the alternative frequency dependent formulation where the force of transmission depends on the proportion of all patients present who are infectious.) Susceptible patients can be infected by several sources while in hospital: infectious patients on the same ward (at a rate β_w_ per infectious patient per day); infectious patients in the same hospital, but on a different ward (β_h_); ward-based environmental contamination left by previous patients on the same ward (detailed below); and a background rate representing infection from unsampled sources, for example unsampled asymptomatic patients (β_bh_). Patients can also become infected in the community, at a community-specific background rate (β_bc_).

As bed-level admission data and detailed hospital layout data are not available, mixing of patients at the level of a ward is assumed to be homogenous such that any infectious patient present on the ward on a given day is equally likely to be the source of a new infection. In support of this, most patients in the dataset studied did not have individual patient rooms, and commodes and bathroom facilities were typically shared at a ward level. Mixing is also assumed to be homogenous at a hospital level as well when considering infections transmitted from patients on other wards, e.g. via visits to common areas such as radiology or via contact with the same healthcare workers.

Patients are only considered infectious while in hospital, from the time interval after they become infected, until the time interval before they recover. While infectious, patients can infect other patients on any day during their hospital stay, including the day of admission and day of discharge.

We allow for persisting contamination of the hospital environment by *C*. *difficile* spores after a patient has left a ward or recovered. Patients are assumed to leave environmental contamination on any ward they are admitted to while infectious. The environmental contamination starts in the time interval after a ward discharge, or the time interval in which a patient recovers. The force of infection from environmental contamination is additive over all contributing patients. Per patient, the initial force of infection from environmental contamination is the same irrespective of the duration of ward stay while infectious and is determined by the product of ϕ_c_ (constrained to be between 0 and 1, representing the relative infectiousness of environmental contamination compared to an infectious patient on the same ward) and β_w_. The level of environmental contamination from each source also decays over time following a geometric distribution (analogous to an exponential distribution in continuous time), governed by a parameter, ω, the proportion of the force of infection lost after each time step.

The time interval between a patient becoming infected and being sampled is assumed to follow a negative binomial distribution (analogous to a gamma distribution in continuous time), with parameters λ_mean_ and λ_size_. Patients are assumed to be infectious during this period, representing a combination of possible transmission from i) pre-symptomatic colonised patients who will go on to display symptoms and ii) from symptomatic patients prior to diagnosis. Weakly informative priors were chosen such that most intervals were expected to be <100 days. Explicitly modelling this interval is adopted to allow the duration of time between acquisition and diagnosis in patients infected by other patients (where the source is sampled and therefore the time of acquisition is relatively constrained by ward overlaps) to inform on the likely time of acquisition in patients infected by background sources in the hospital and community (where the source is not sampled nor the time of transmission otherwise constrained). If this interval is estimated to be long this will favour acquisition from community-based background sources, conversely if it is short, hospital-based background sources are more likely.

Similarly, the time interval between a patient being sampled and recovering, i.e. ceasing to be infectious, is also assumed to follow a negative binomial distribution, with parameters γ_mean_ and γ_size_. The timing of sampling is assumed to determine the start of recovery as only patients diagnosed with an infection are considered and these patients receive treatment following sampling. Weakly informative priors were chosen to set the median recovery time to around 12 weeks (in keeping with previous empirical observations [17]), but allowing for shorter times, and times up to a year.

For computational tractability, patients who are not sampled and patients with only negative tests for infection are assumed not to be cases, i.e. the diagnostic test is assumed to be perfectly sensitive. Allowing for this simplifying assumption, as diagnostic testing for *C*. *difficile* is in fact imperfectly sensitive, transmission events arising from any unsampled or falsely negative patients, as well as those asymptomatically colonised without ever developing infection, are attributed to the hospital background source. The formulation of the model also means that there will be patients who are asymptomatically colonised with *C*. *difficile*, but who do not go on to become infected, who are labelled susceptible, as the model only considers acquisition that leads to a subsequent infection.

Transmission model parameters are summarised in Table 1.

**Table 1:** Transmission model parameters and priors. For parameters constrained to be positive, normal priors were truncated at zero. In addition to the listed parameters, augmented data included the unobserved transmission tree, consisting of infection times and sources denoted *T* and the set of unobserved recovery times denoted *R*.

Model component	Parameter	Description	Prior	Justification
Transmission model	α	Probability already infected at the start of the study period, i.e. time, t = 0 (constrained to between 0 and 1 by logistic transformation)	Normal(mean = -8, sd = 1), on untransformed scale	Assuming that the probability of starting infected is low, i.e. ~1 in 3000, reflecting annual incidence of ~1 in 1000 in the at risk population, and duration of ~100 days[18]
	β_bh_	Daily rate of transmission from unsampled sources while in hospital (asymptomatic carriers, environment, etc.)	Gamma(shape = 2, scale = 0.002)	Arbitrary, but subsequent posterior estimates substantially different (e.g. S15 Fig)
	β_bc_	Rate of transmission per day spent in the community from any source	Gamma(shape = 2, scale = 0.002)	
	β_w_	Daily rate of transmission per infected individual on the same ward	Gamma(shape = 2, scale = 0.002)	
	β_h_	Daily rate of transmission per infected individual on other wards	Gamma(shape = 2, scale = 0.002)	
	ϕ_c_	The relative infectiousness of ward contamination compared to an infectious individual on the same ward (constrained to between 0 and 1 by logistic transformation)	Normal(mean = 0, sd = 1.7), on untransformed scale	Minimally informative between 0 and 1 on transformed scale
	ω	Geometric parameter determining the rate of decline of ward contamination (constrained to between 0 and 1 by logistic transformation)	Normal(mean = 0, sd = 1.7), on untransformed scale	Minimally informative between 0 and 1 on transformed scale
	λ_mean_ and λ_size_	Negative binomial parameters determining the time between infection and sampling	Gamma(shape = 3, scale = 10) and Gamma(shape = 0.7, scale = 2)	See Methods text
	γ_mean_ and γ_size_	Negative binomial parameters determining the time between sampling and recovery	Normal(mean = 90, sd = 15) and Normal(mean = 3, sd = 0.5)	
Overall	*θ*	Summary notation for all parameters.		

### Inference overview

We use a similar representation to Worby *et al* [4] for the likelihood of the observed data for a given set of transmission parameters. We extend this approach to include recovery from infection and multiple routes of transmission in hospital as above. Additionally, we present an alternative formulation for the genetic component of the likelihood.

We denote the observed set of sample collection dates *E* and the observed sequencing data *S*. The augmented unobserved transmission tree, consisting of infection times and sources is denoted *T* and the set of unobserved recovery times *R*. The likelihood of these observed and augmented data given our parameters, *θ*, can be factorized as follows:

Pr(S,E,T,R|θ)=Pr(S|E,T,R,θ)Pr(E,T,R|θ)

As the sequencing data, *S*, does not depend on the recovery times, *R*, this simplifies to:

Pr(S,E,T,R|θ)=Pr(SE,T,θ)Pr(E,T,R|θ)

Taking each element of the likelihood in turn, Pr(*E*, *T*, *R*|*θ*) represents the transmission model, which is described in more detail below. Pr(*S*|*E*, *T*, *θ*) is the likelihood of the genetic data for a given transmission tree and sampling dates.

### Transmission model likelihood

To determine Pr(*E*, *T*, *R*|*θ*), we first consider a single patient, *i*, who is not infected at the start of the study (with probability (1−*α*)), who becomes infected at $t_{i}^{infect}$. The likelihood of their infection time, $t_{i}^{infect}$, source of infection, sampling time, $t_{i}^{samp}$, and recovery time, $t_{i}^{rec}$, given the parameters *θ*, is the product of: the probability of avoiding infection until $\left(t_{i}^{infect}-1\right)$; the probability of being infected at $t_{i}^{infect}$; the probability of the specific source of their infection given infection at $t_{i}^{infect}$; the probability of being sampled at $t_{i}^{samp}$ given $t_{i}^{infect}$; and the probability of recovering at $t_{i}^{rec}$ given $t_{i}^{samp}$.

The probability of avoiding infection until $\left(t_{i}^{infect}-1\right)$ is given by:

$$\mathrm{Pr}(\mathrm{avoid}\;\mathrm{infection}\;\mathrm{until}\;t_{i}^{infect}-1|\theta )=\exp\left(-\sum_{t=0}^{t_{i}^{infect}-1}B_{i}^{t}\right)$$

where $B_{i}^{t}$ is the total force of infection experienced by a particular patient, *i*, at time *t*.

Patients experience a constant background rate of infection from unsampled sources. When a patient is located in the hospital this is given by β_bh_ and when their location is the community by β_bc_. For a patient, *i*, admitted to hospital the force of infection at time *t* is determined by the ward within the hospital they are admitted to, denoted *a*. $I_{w}^{a}(t)$ is the number of infectious patients on ward *a* at time *t*, $I_{h}^{a}(t)$ is the number of infectious patients on other wards in the same hospital (multiple hospitals are present in the dataset), and $C_{w}^{a}(t)$ is the level of environmental contamination on ward *a*, giving:

$$B_{i}^{t}=\left\{ \begin{array}{l} \;\beta_{bc},location=community\\ \beta_{bh}+\beta_{w}I_{w}^{a}(t)+\beta_{h}I_{h}^{a}(t)+\beta_{w}\phi_{c}C_{w}^{a}(t),location=ward\;a \end{array}\right.$$

ϕ_c_ denotes the relative infectiousness of ward contamination compared to an infectious individual on the same ward (constrained to between 0 and 1). The level of ward contamination at time *t*, $C_{w}^{a}(t)$, is determined by the *τ*(*t*) patients who have been previously discharged from the same ward, *a*, while still infectious or have recovered on the ward. The contribution of each such patient, *p*, to the total environmental contamination at time *t* declines geometrically with increasing time between $t_{c}^{a}(p)$, the time of discharge or recovery of patient *p* and *t*:

$$C_{w}^{a}(t)=\sum_{p=1}^{\tau (t)}({1-)}^{\left(t-t_{c}^{a}(p)\right)}$$

ω is a geometric parameter determining the rate of decline of ward contamination.

The probability of patient *i* being infected by any source at $t_{i}^{infect}$, conditional on being uninfected at $t_{i}^{infect}-1$ is:

$$\mathrm{Pr}(\mathrm{infected}\;\mathrm{at}\;t^{infect}|\theta )=1-\exp\left(-B_{i}^{t^{infect}}\right)$$

When we come to evaluate the genetic support for a transmission event a specific source of transmission needs to be proposed. Therefore, we include individual sources of infection in the transmission model. We define the probability, *ζ*_i_, of patient *i* being infected by a specific source patient or background source, given patient *i* became infected at $t_{i}^{infect}$, based on the route of transmission as in Table 2.

**Table 2:** Probability of infection by a given source patient or background source.

Location	Source type	Probability of a specific source	Source
Community	Community background	1	Background
Hospital	Hospital background	$\beta_{bh}/B_{i}^{t^{infect}}$	Background
	Ward	$\beta_{w}/B_{i}^{t^{infect}}$	Specific patient
	Hospital	$\beta_{h}/B_{i}^{t^{infect}}$	Specific patient
	Ward contamination	$\beta_{w}\phi_{c}({1-\omega )}^{\left(t_{i}^{infect}-t_{c}^{a}(p)\right)}/B_{i}^{t^{infect}}$	Contamination arising from a specific patient, *p*

For a patient who is never infected, the probability of avoiding infection is calculated as above, but now summing over all time considered (*t* = 0 to *t* = *t*^*max*^).

The probability of a patient *i* being sampled at $t_{i}^{samp}$ is defined relative to their infection time, $t_{i}^{infect}$, such that the probability of being sampled $\left(t_{i}^{samp}-t_{i}^{infect}\right)$ days after infection is given by the probability mass function, *f*, for the negative binomial distribution with parameters λ_mean_ and λ_shape_, denoted *κ*. Therefore, for all *n* infected patients,

$$\prod_{i=1}^{n}f(t_{i}^{samp}-t_{i}^{infect})=\prod_{i=1}^{n}\kappa_{i}$$

Similarly, the probability of recovering at $t_{i}^{rec}$ is defined relative to the sampling time, $t_{i}^{samp}$. The probability of recovering ${(t}_{i}^{rec}-t_{i}^{samp})$ days after sampling is given by the probability mass function, *g*, for the negative binomial distribution with parameters γ_mean_ and γ_shape_, denoted *ψ*. Therefore,

$$\prod_{i=1}^{n}g(t_{i}^{rec}-t_{i}^{samp})=\prod_{i=1}^{n}\psi_{i}$$

Overall the likelihood Pr(*E*, *T*, *R*|*θ*), including those not infected, is the product of these terms above, for the set M of *m* patients who remain uninfected at *t*^*max*^ and the set N of *n* infected patients, *q* of whom start infected:

$$\begin{array}{l} \mathrm{Pr}(E,T,R|\theta )=\alpha^{q}(1-\alpha {)}^{(m+n)-q}\\ \;\times \prod_{i\in N}(1_{t_{i}^{\mathrm{infect}}<0}\\ \;+\left(1_{t_{i}^{\mathrm{infect}}\geq 0}\exp\left(-\sum_{t=0}^{\max\left(0,t_{i}^{\mathrm{infect}}-1\right)}B_{i}^{t}\right)\zeta_{i}\left(1-\exp\left(-B_{i}^{t_{i}^{\mathrm{infect}}}\right)\right)\right) \end{array}$$

$$\times \prod_{j\in M}\exp\left(-\sum_{t=0}^{t^{max}}B_{j}^{t}\right)\times \prod_{i\in N}\kappa_{i}\psi_{i}$$

Where $1_{t_{i}^{infect}<0}$ is an indicator variable taking the value 1 where the specified condition is true, and zero otherwise.

### Genetic model and likelihood

Assessing the likelihood of a collection of genomes conditional on the underlying transmission tree is very challenging [4–7], the more so given the presence of recombination [19,20]. To make progress, we adopted an approximate approach that sought to exploit the valuable information about transmission while achieving computational efficiency and avoiding artefacts caused by recent recombination.

Conceptually, we evaluated the joint likelihood of the observed genomes given the transmission events and other parameters. In practice, we defined the approximate likelihood so that it depended only on an *n*×*n* pairwise genetic distance matrix *d*, distances which we inferred by estimating a recombination-corrected maximum likelihood phylogeny using PhyML[21] and ClonalFrameML [19].

The structure of our approximate likelihood was a composite or quasi-likelihood of the form

$$Pr(S|R,E,T,\theta )\approx \prod_{i=1}^{n}\mathrm{Pr}\left(S_{i}|\{S_{k}:k\neq i),R,E,T,\theta \right)$$

Here we consider the likelihood of each sequence, *S*_*i*_, given all other sequences, denoted *S*_*k*_. We approximated each term in the likelihood depending on whether the source of infection was another case versus background.

### Infection from another case

**Fig 2:**
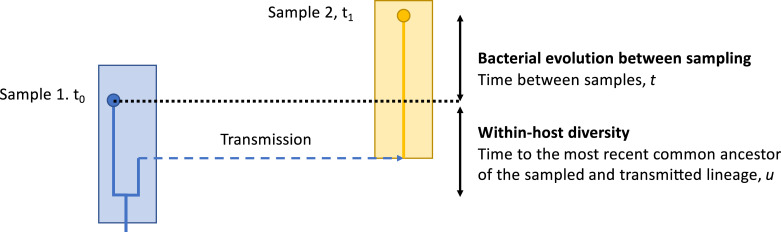
Coalescent-based model. Each box represents an infected patient (blue: patient *j*, yellow: patient *i*). Time runs from the bottom of the plot to the top. Samples are shown as circles. *t*_0_ denotes the time of the first sample, *t*_1_ the time of the second sample. *t* is the time between the samples, and *u* the time between the first sample and the common ancestor of the samples.

When the source of infection of case *i* was patient *j*, we approximated the conditional likelihood by considering only the integer genetic distance between the two genomes, *d*_*ij*_. This distance is determined by the extent of with-host diversity in the transmission source, the rate of mutation (*μ* per unit time), and time *t* between their samples. Based on coalescent theory, and assuming mutations are rare with respect to the length of the genome (known as the infinitely many sites assumption), the pairwise likelihood depends only on the genetic distance between the two genomes. This can be described as the sum of two Poisson processes, firstly the variation that arose in the time, *t*, between the samples being taken, and secondly the variation that has arisen in the time, *u*, between the time of the most recent common ancestor of the two samples and the time the first sequence was obtained, *t*_0_ (Fig 2).

We make the simplifying assumption that we can ignore the genetic bottleneck that founded the infection in the source patient *j* and assume the source had bacterial effective population size *N*_*e*_. If *u* were known then, by the infinitely many sites assumption, we would model *d*_*ij*_ using a Poisson distribution with mean equal to the mutation rate *μ* multiplied by the total time for mutations to occur (*t*+2*u*):

$$d_{ij}∼Pois(\mu (t+2u))$$

Since *u* is not known we integrate over its possible values. We approximate by assuming a standard coalescent process with effective population size *N*_*e*_, which implies that:

$$u∼Exp(\frac{1}{N_{e}})$$

The probability of a given SNP distance conditional on *u* is, from the Poisson distribution:

$$\mathrm{Pr}\left(d_{ij}|u,t,\mu \right)=\frac{e^{-\mu (t+2u)}{\left(\mu (t+2u)\right)}^{d_{ij}}}{d_{ij}!}$$

The probability density of *u* is, from the exponential distribution:

$$p(u|N_{e})=\frac{1}{N_{e}}e^{-\frac{1}{N_{e}}u}$$

Integrating over all possible values of *u* gives:

$$\mathrm{Pr}\left(d_{ij}|N_{e},t,\mu \right)=\int_{0}^{\infty }\mathrm{Pr}\left(d_{ij}|u,t,\mu \right)p(u|N_{e})du$$

$$=\frac{e^{-\mu t}}{N_{e}d_{ij}!}\int_{0}^{\infty }{\left(\mu (t+2u)\right)}^{d_{ij}}e^{-u(2\mu +\frac{1}{N_{e}})}du$$

$$=\frac{e^{\frac{t}{2N_{e}}}\Gamma (1+d_{ij},\mu t+\frac{t}{2N_{e}})(2{\mu )}^{d_{ij}}}{N_{e}d_{ij}!{\left(2\mu +\frac{1}{N_{e}}\right)}^{d_{ij}+1}}$$

where $\Gamma (1+d_{ij},\mu t+\frac{t}{2N_{e}})$ is an incomplete gamma function.

Therefore we assume

$$\mathrm{Pr}(S_{i}|\{S_{k}:k\neq i\},R,E,T,\theta )\propto \mathrm{Pr}\left(d_{ij}|N_{e},t,\mu \right).$$

where *N*_*e*_ and *μ* are contained within *θ* and *t* depends on the sampling times (*E*), and *i* and *j* are drawn from the transmission tree (*T*).

### Infection from background

The other possible scenario is where a case is inferred to have been acquired from a hospital or community background source, i.e. the immediate source has not been sampled. We approximated the conditional likelihood by considering only the integer genetic distance *d*_*ij*_ between the genome of case *i* and another case *j*, assumed to be sampled at random from the population. For a random member of the population, not related by recent transmission, the coalescence time is likely to be much longer, to the extent that time differences between samples are assumed to be negligible compared to the age of the population as a whole. The distribution of coalescence times was parameterized by *N*_*pop*_, the effective size of the background population.

If the time, *v*, to most recent common ancestor of a pair of samples *i* and *j* were known then, by the infinitely many sites assumption, we would model their genetic distance, *d*_*ij*_, with a Poisson distribution with mean equal to the total time for mutations to occur, 2*v*, multiplied by the mutation rate *μ*:

$$d_{ij}∼Pois(2v\mu )$$

Since *v* is not known we integrate over its possible value. We approximate by assuming a standard coalescent process with effective population size *N*_*pop*_, which implies that:

$$v∼Exp(\frac{1}{N_{pop}})$$

Integrating over *v* gives:

$$\mathrm{Pr}\left(d_{ij}|N_{pop},\mu \right)=\int_{0}^{\infty }\mathrm{Pr}\left(d_{ij}|v,\mu \right)p(v|N_{pop})dv$$

$$={\left(\frac{2N_{pop}\mu }{2N_{pop}\mu +1}\right)}^{d_{ij}}\frac{1}{2N_{pop}\mu +1}$$

which is a geometric distribution with parameter $p=\frac{2N_{pop}\mu }{\left(2N_{pop}\mu +1\right)}$, probability $\mathrm{Pr}\left(d_{ij}|p\right)=p^{d_{ij}}(1-p)$ and mean 2*N*_*pop*_*μ*, which is the expected diversity of randomly sampled sequences from the population.

Since case *j* was chosen at random, we next averaged over the possible identity of case *j*. Formally, we adopted a quasi-likelihood of the form:

$$\mathrm{Pr}(S_{i}|\{S_{k}:k\neq i\},R,E,T,\theta )\propto {\left(\prod_{j\neq i}\mathrm{Pr}\left(d_{ij}|p\right)\right)}^{\frac{1}{n-1}}$$

Similar to above, *N*_*pop*_ and *μ* on which *p* depends are contained within *θ* and the pool of *n* cases from *i* and *j* are drawn is specified by the transmission tree (*T*). We took a geometric mean rather than an arithmetic mean because this gave superior performance in preliminary simulations. While our genetic likelihoods were highly approximate, we subjected the method to rigorous testing to establish satisfactory performance in detailed simulations as detailed in the first section of the Results.

Table 3 provides a summary of the genetic model parameters.

**Table 3:** Genetic model parameters and priors. For parameters constrained to be positive, normal priors were truncated at zero.

Model component	Parameter	Description	Prior	Justification
Genetic model	*μ*	Mutation rate per genome per day	Normal(mean = 0.8/365.25, sd = 0.295/365.25)	Point estimate and confidence interval from [2]
	*N*_*e*_	Effective bacterial population size within an infected patient	Normal(mean = 74.0, sd = 18.5)	Point estimate and confidence interval from [2]
	*N*_*pop*_	Effective bacterial population size with the population reservoir	Gamma(shape = 2, scale = 10000)	Represents mean pairwise diversity between randomly chosen representatives of a sequence type of ~100 SNPs.
Overall	*θ*	Summary notation for all transmission and genetic model parameters: {*α*, *β*_*bh*_, *β*_*bc*_, *β*_*w*_, *β*_*h*_, *ϕ*_*c*_, *ω*, *λ*_*mean*_, *λ*_*size*_, *γ*_*mean*_, *γ*_*size*_, *μ*, *N*_*e*_, *N*_*pop*_}		

### Markov chain Monte Carlo algorithm and data augmentation

Samples are drawn from π(*θ*|*E*, *S*) using MCMC, also integrating over all possible values of the augmented variables *T* and *R*, as by Bayes theorem:

$$\pi (\theta |S,E)\propto \int_{T,R}\pi (S,E,T,R|\theta )\pi (\theta )$$

Initial infection times and recovery times are set by randomly sampling values relative to the sampling time from two negative binomial distributions, determined by the starting values of λ_mean_ and λ_shape_ and γ_mean_ and γ_shape_. Initially the source of all infections is set to be background, with a community or hospital route depending on the patient’s location at the sampled infection time. Where the sampled infection time is before t = 0, the patient is initially set to have started infected.

The MCMC algorithm proceeds in three stages per iteration, 1) parameter updates, 2) one-at-a-time infection time, source and recovery time updates, 3) block updates to infection times and sources (summarised in S1 Fig).

The parameters are updated one at a time, using a univariate normally distributed proposal centred on the current value. If the proposed parameter value is negative, except for logistic transformed parameters, the proposal is rejected. The variance of the proposal distribution is adapted during the initial part of the chain to target a proportion of accepted proposals between 0.20 and 0.40 and the adaptive phase discarded as part of the burn-in period.

For each iteration, following proposals/updates for each of the parameters, 10% of cases are selected at random. For each selected case, updated infection times are proposed one at a time from a normal distribution centred on the current value. The standard deviation of the distribution can take either a small value (5 days) or large value (25 days) with 50% probability to allow for moves of different sizes. If a proposed infection time is not compatible with the existing transmission tree the move is rejected, i.e. because the new infection time is after an onward transmission, or after a ward discharge that has left contamination leading to onward transmission. Based on the proposed infection time, a source of the infection is drawn. The relative probability of each source is determined by the product of their relative infectiousness (see Table 2) and their pairwise genetic likelihood. The proposed new infection time and source together are then compared to the current state. As the change in infection time is symmetrically proposed, with equal probabilities of large and small moves, this does not itself contribute to the Hastings ratio, which is calculated as the probability of choosing the current source, given the current infection time divided by the probability of choosing the proposed source, given the proposed infection time.

Updates to recovery times are made in a similar way, one-at-a-time for cases in the 10% chosen, proposing an updated time from a normal distribution centred on the current time (standard deviation 25 days), rejecting moves not compatible with the transmission tree, i.e. moving the recovery time before an onward transmission, moving the recovery time before a ward discharge that leaves contamination that leads to onward transmission, or moving the recovery time later such that an existing transmission via contamination can no longer occur.

Finally, at each iteration, one block update of infection times and sources is proposed. This is to allow reversal of the direction of transmission, which is only possible when the sources of two or more infections are updated simultaneously. A case, *z*, is selected at random, all cases that have *z* as their direct or indirect source are identified following the edges of the transmission tree, until a maximum limit is reached of 10 cases. For case z and the subsequent cases together, the set of cases Z, all transmission events leading to these cases are removed from the list of constraints on updated infection times. New infection times are proposed for each of the cases in Z. Then working forwards in time, new sources are sampled as above for each case, each conferring a component to the overall Hastings ratio. The acceptance ratio is then calculated for the new infection times and sources as a whole.

Parameter priors, π(θ), are given in Tables 1 and 3. Default values assume the rate of mutation and extent of within host genetic diversity are previously well described, hence the relatively tight priors which match previous findings [2].

For each analysis at least 3 separate chains of at least 50000 iterations were run and evaluated to confirm convergence. Chains were visually inspected to set an appropriate burn-in of ≥20%. Convergence was confirmed using Gelman-Rubin-Brooks plots, generated using the R coda library, and requiring scale reduction factors of ≤1.1 to be achieved for each parameter. After discarding the burn-in period, all chains were combined, including sufficient iterations to achieve an effective sample size of ≥200 for all parameters.

C++ code implementing our model is available at https://github.com/davideyre/transmission_inference.

### Simulations

We used simulation to assess the performance of our model by comparing model inferences based on simulated ward movements, sample dates and genomic data, with the known underlying simulated transmission events and parameters. Fifty simulations, 10 from each of 5 scenarios were generated using a simulated community of individuals admitted to a hospital at random, using the transmission model described above. The 5 scenarios varied in complexity (Table 4), the simplest included transmission in hospital from infected patients on the same ward and from other sources in the hospital at a constant rate, denoted hospital background above. The most complex also allowed for spore-based transmission arising from persistent ward contamination after discharge or recovery of a case, transmission across different hospital wards, and a community-based background rate of transmission. Performance under different durations of spore persistence was tested (“short” and “long” duration spores). Genetic data were simulated using the multi-species coalescent as described previously [22] with a narrow simulated bottleneck at transmission. Simulation parameters were chosen to represent a population at relatively high risk of admission to hospital and contracting *C*. *difficile*, i.e. typical of a cohort of patients that might be at risk of admission to an acute medical ward. The intervals between acquisition and sampling and sampling and recovery were chosen to be similar to the priors provided above, i.e. consistent with previous literature. Transmission parameters were chosen such that the number of infections acquired by each route of transmission simulated typically exceeded 10–20 cases.

**Table 4:** Simulation scenarios. Simulated scenarios for a hospital with 4 wards, serving a population of 6000 patients at risk of admission with a daily probability of admission of 0.002 and a mean length of stay of 5 days. Simulation run for 365 days. Sampling distribution parameters, μ = 10, size = 5; Recovery distribution parameters, μ = 90, size = 3. Assuming 1 mutation per genome per year, *N*_*pop*_ = 20000 and *N*_*e*_ = 22.5. All patients assumed to start susceptible.

Scenario	β_bh_	β_w_	β_h_	β_bc_	ω	ϕ_c_
Ward only	0.002	0.005				
Ward + hospital	0.002	0.005	0.001			
Ward + hospital + short duration spores	0.002	0.005	0.001		0.3	0.9
Ward + hospital + long duration spores	0.002	0.004	0.001		0.1	0.5
Ward + hospital + spores + community	0.001	0.003	0.001	0.00003	0.2	0.8

We compared the performance of our model to two alternatives. The first was to fit the same transmission model but without genomic data to investigate the added value of sequencing data. The second was a heuristic algorithm for assigning the source of infection similar to one we have used in previous descriptive studies [2,17]: initially all prior cases within ≤2 SNPs of a case were considered potential sources and ranked as follows, accounting for epidemiological links: potential sources with shared time on the same ward as the case, following the source’s diagnosis and prior to the case’s, were ranked highest. Cases admitted to the same hospital ward, within ≤28 days following the discharge of an infected source, were ranked next, and finally cases sharing time in the same hospital, but not in the same ward were ranked last. Within each class of transmission route, the most plausible source was chosen as the source diagnosed closest in time to the diagnosis of the case. Similar to *C*. *difficile* surveillance recommendations [23], cases >2 SNPs from any previous case, or without an epidemiological link to another case ≤2 SNPs, were classified as acquired from hospital background if diagnosed within 12 weeks of a hospital stay, and community acquired if >12 weeks since the last hospital exposure (or if no previous hospital exposure).

We conducted additional simulations to test if our model was robust to variation in the transmission parameters by setting the daily probability of hospital admission to 0.02 to increase the number of patients in hospital, but reducing *β*_*bh*_, *β*_*w*_, *β*_*h*_ to 2e-4, 2e-4 and 3e-5 respectively and setting *β*_*bc*_ to zero and *ω* and ϕ_c_ to 0.2 and 0.8. We also undertook simulations to assess the impact of reducing the sample size, and therefore power, setting *β*_*bh*_, *β*_*w*_, *β*_*h*_ to 0.002, 0.005 and 0.001 respectively and setting *β*_*bc*_ to zero and *ω* and ϕ_c_ to 0.2 and 0.8, we generated 10 simulations each for population sizes of 6000, 5000, 4000, 3000, 2000, and 1000 individuals.

### *C*. *difficile* transmission in Oxfordshire

We applied our model to a previously published whole-genome sequencing study of consecutive *C*. *difficile* infections in Oxfordshire, UK, from 2007–2011 [2]. Sequences were available from 1223 CDI cases, together with dates of sampling. Data on ward admissions and discharges for all patients, cases and non-cases, were available for the study period. Separate analyses were performed for infections from each of the 10 most prevalent subtypes of *C*. *difficile*, based on MLSTs determined previously [2]. This approach allowed differences in transmission properties of different sequence types (STs) to be investigated and also allowed for separate estimates of *N*_*pop*_ per ST, reflecting the differing levels of genetic diversity present within each ST. To generate aggregate findings across all STs, 10,000 samples were obtained from each ST’s final combined MCMC chain. For each iteration counts of the transmission events of each type were combined across STs, and the posterior mean and highest posterior density (HPD) interval calculated. We used previous estimates of *C*. *difficile* mutation rates and the extent of within-host diversity [2] to set relatively tight priors for *μ* and *N*_*e*_, rather than expect our model to estimate these parameters from the data.

## Results

### Simulations demonstrate our model is accurate and well calibrated

We used simulated data to assess the accuracy of inferences made using our transmission model. Fifty simulations were generated, 10 from each of 5 transmission route scenarios. In the simplest scenario, in which transmission could arise from other cases present on the same ward or at a constant background rate for patients admitted to the hospital (reflecting unmeasured sources of in-hospital transmission), the median (range) percentage of true source types (ward vs. background) correctly estimated as the most likely was 92.4% (83.3–96.6%), and the percentage of correct individual sources was 86.5% (65.0–94.9%).

In an alternative scenario allowing for transmission via one of five routes (hospital background, community background, ward, spore and hospital-wide transmission) the percentages were 83.9% (80.0–92.3%) and 84.4% (76.6–91.3%) respectively. The higher value for the correct individual source reflects that the correct source patient could be identified but with the wrong transmission route, e.g. ward vs. spore-based transmission.

**Fig 3:**
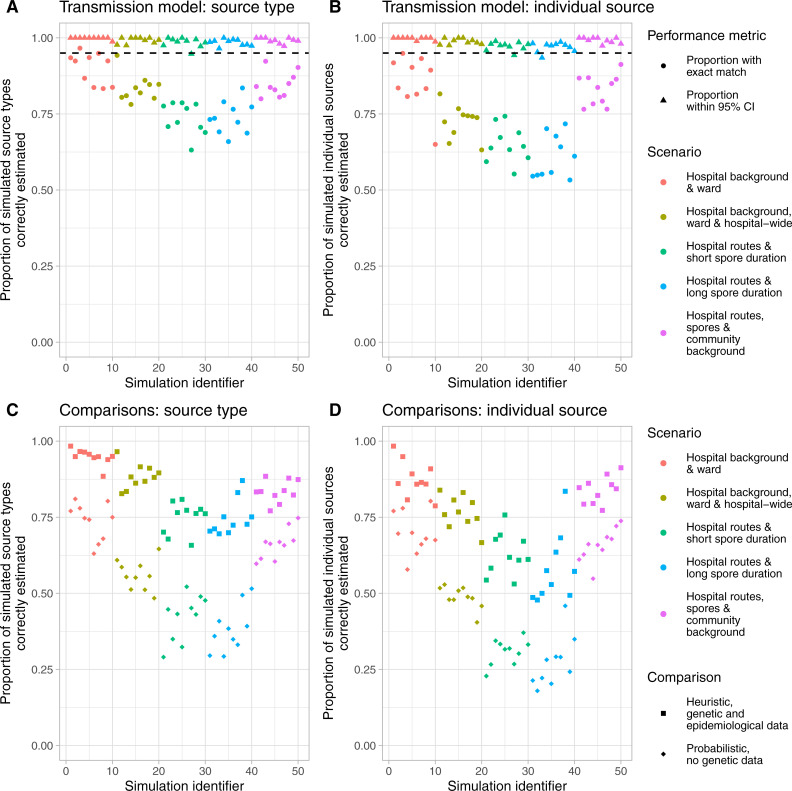
Source type and individual source attribution in simulated data. The left-hand columns (panels A and C) display the accuracy of inferences about the source type, i.e. distinguishing between hospital background, community background, ward, spore and hospital-wide transmission. The right-hand columns (panels B and D) display the accuracy of inferences about the specific patient source of each infection (for background source types this is an unknown source). Panels A and B display inferences from the full transmission model. Panels C and D display the accuracy of predictions running an inference model without using genetic data (circles) or based on a heuristic rule (squares). CI, credibility interval.

Results from these and other scenarios are shown in Fig 2. Uncertainty in the estimates was well calibrated, with the 95% highest posterior probability sets (95% credibility intervals, CI) capturing the true source type >95% of the time in 49/50 (98%) simulations and true individual sources >95% of the time in 48/50 (96%) of simulations (Fig 3A and 3B).

**Fig 4:**
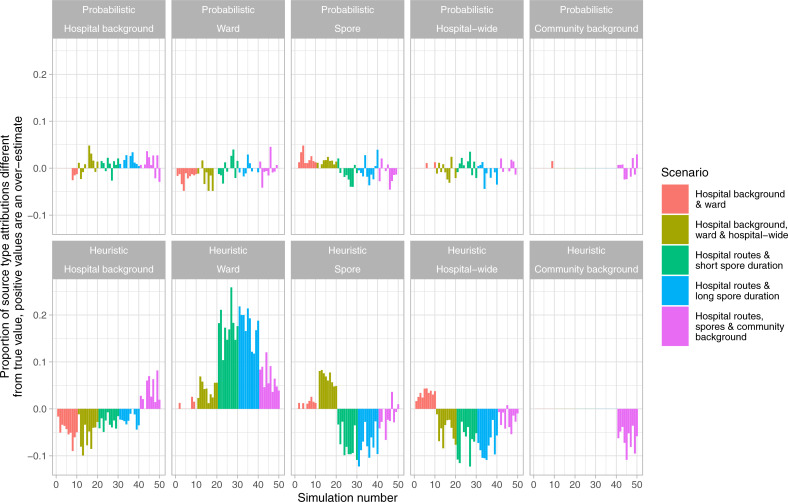
Comparison of source type attribution using a stochastic transmission model and heuristic rules.

Similar proportions of simulated source types and individual sources were correctly identified using a heuristic transmission rule that assigned the single most likely source of each case based on genetic similarity and temporospatial proximately of cases, in the simplest scenario 95% (88.5–98.4%) and 86.3% (78.8–98.4%), and in the scenario with five routes 83.4% (77.1–88.5%) and 84.5% (77.3–91.3%) respectively (Fig 3C and 3D). In contrast, the performance of the transmission model without genetic data was substantially worse (Fig 3C and 3D). However, estimates from the heuristic transmission rule showed consistent biases that were not present in estimates from the transmission model using genetic and epidemiological data (Fig 4). The heuristic rule contains a hierarchy of transmission events, with ward-based transmission favoured over transmission via spores left behind after discharge/recovery or hospital-wide transmission. This resulted in consistent over-estimates of the extent of transmission via direct contact on the same ward, but under-estimates of the extent of transmission via spores or across hospital wards.

Further details of the precision of source type attribution within each scenario are provided in S2–S6 Figs. Details of the accuracy of inferred infection times and parameter estimates are provided in S7 and S8 Figs. We also undertook a sensitivity analysis of the performance of our model with lower rates of transmission, which demonstrated model estimates remained accurate and uncertainty estimates well-calibrated (S9 Fig). We also simulated the effect of reduced sample size on the power of our model to estimate transmission parameters (S10 Fig, S1 Table), which suggested that around 40–70 cases were sufficient to estimate the main transmission parameters.

### Oxfordshire *C*. *difficile* infection–inference demonstrates strain-specific differences in transmission

Isolates from 1223 of 1250 consecutive hospital and community onset CDI cases between 01 September 2007 and 31 March 2011 in Oxfordshire, UK, underwent whole-genome sequencing as previously described [2]. Linked epidemiological data on all patient admissions and ward movements, for cases and non-cases, at the county’s four hospitals were available. All isolates in the study also underwent MLST, the incidence of each ST in the study and temporal trends in STs are shown in S11 Fig. The most notable change was in ST1, which was the most common ST overall, but declined markedly during the study, such that only 2 ST1 CDIs were seen in the final year of the study, i.e. after 31 March 2010. S12 Fig shows a maximum likelihood phylogeny of the 1223 *C*. *difficile* genomes studied. *C*. *difficile* follows a conserved clade structure, with 5 major clades, and within these clades the large majority of genomes from different STs form conserved sub-clades. Therefore, to account for differences in the transmission of different *C*. *difficile* lineages and allow comparisons between lineages in a way compatible with previously published literature, 11 separate models were fitted for the 10 most common STs, and the remaining STs as a single group. Although the group of “Other” STs is genetically diverse, the fact that these STs are relatively less frequent reflects that they share in common more limited healthcare-associated transmission. We therefore analysed these lineages as a single group as we lacked sufficient numbers of cases to analyse each separately. The extent of genetic diversity within each ST is summarised in S13 Fig.

### Routes of acquisition

Summarising the findings using posterior means, of the 1223 cases, 262 (21% [95% CI 20–22%]) were estimated to have been acquired from another known case: 121 (10% [9–11%]) via contact during overlapping ward stays, 96 (8% [6–9%]) by spores left behind after discharge or recovery, and 46 (4% [3–5%]) via contact during overlapping stays in different wards. The remaining 950 (78% [77–79%]) cases were estimated to be acquired from other unmeasured sources of transmission. Based on the assumption that the time between acquisition and sampling was similar in hospital to the community, then 819 (67% [66–68%]) were attributed to hospital-background and 131 (11% [10–11%]) to the community. However, given there are several possible reasons that the time interval between acquisition and sampling may vary by patient location, this breakdown by hospital versus community should be interpreted with caution and the headline figure of 78% [77–79%] of cases attributed to unmeasured sources regarded as the main result.

**Fig 5:**
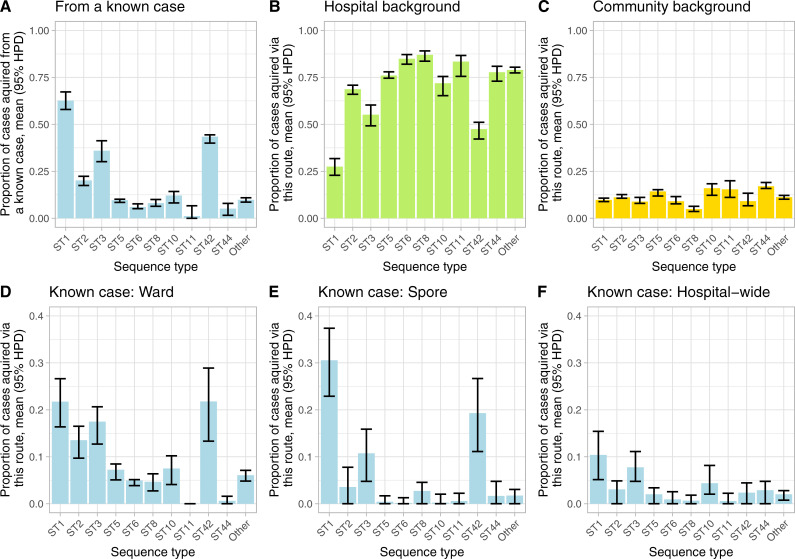
Proportion of cases acquired from each transmission route, by sequence type. Panels A to C show the estimated proportion of cases acquired from each of the 3 major source types, known patients (A, blue), hospital background sources (B, green) and the community (C, gold). Panels D to F subdivide the cases shown in panel A, into those acquired by direct ward contact (D), ward-based spores (E) and hospital-wide transmission (F). Note the y-axis scale differs between the upper panels and lower panels, to allow for comparison of the relative contribution of ward, spore and hospital-wide routes to transmission from other cases, and hospital vs. community for background transmission.

There was heterogeneity by ST in the proportion of cases acquired from a known case, which was highest in ST1 (most common ribotype equivalent: 027), 63% (131.2/214 cases, 95%CI 58–67%), ST42 (ribotype-106), 43% (19.6/45 cases, 40–44%), and ST3 (ribotype-001), 36% (22.7/63 cases, 30–41%), but <10% in most other STs (Fig 5A and 5B). All three ribotypes (027, 106, and 001) have been associated with healthcare-associated outbreaks [24], with ribotype-027 in particular regarded as an epidemic strain [25].

The proportion of cases acquired in the community were similar by ST (Fig 5C), but highest for ST10/44 (ribotype-015) and ST11 (ribotype-078). Sequence types associated with higher rates of transmission from known cases (ST1, ST3 and ST42) as well as having the greatest proportion of cases acquired by direct-ward contact, had higher proportions of cases arising from spores, i.e. residual ward contamination left after discharge or recovery of a case, 31% (23–37%), 11% (5–16%), 19% (11–27%) respectively (Fig 5D–5F).

### Parameter estimates, by ST

Parameter estimates by ST are shown in the Supplementary Materials (S14 Fig shows a example set of MCMC traces, S15–S24 Figs provide summary plots for all parameters and distributions for the transmission parameters). The point estimates for the transmission rate (per 10,000 person bed-days) from other cases present on the same ward, *β*_*w*_, were highest for ST1 (2.5 [95%CI 1.6–3.5]), ST3 (2.6 [1.1–4.1]), and ST42 (3.1 [1.4–5.1]). The rate was significantly higher in ST1 than ST6 (1.2 [0.3–2.2], p = 0.03), ST8 (1.1 [0.4–1.8], p = 0.01), ST44 (0.7 [0.05–1.5], p = 0.005), and the other ST group (1.1 [0.6–1.7], p = 0.004), however the relatively wide credibility intervals shown in S15 Fig reflect the limited power of the study to differentiate between STs.

S20–S22 Figs show the parameters governing the persistence and relative infectiousness of spores. Spores from ST1 persisted significantly longer than those from all other STs other than ST3, ST42 and the other group (all p<0.048) (also see S25 Fig). The mean (95%CI) spore persistence time for ST1 was 39.0 (22.7–66.8) days, compared to 40.2 (5.6–113.9) days for ST3, 22.5 (10.5–45.3) days for ST42, 17.5 (1.2–42.6) days for the other ST group and between 2.5 and 12.5 days for the remaining STs. There was no clear information in the dataset on the relative infectiousness of spores compared to direct ward contact with a case, all estimates were similar to the broad prior (S10 Fig). S23 Fig shows estimated within-host diversity and mutation rates followed the priors set based on a previous study [2] and estimates of within ST diversity reflected the observed distributions of pairwise SNP differences (S13 Fig).

S24 Fig shows the distribution of the parameters governing the time delay between infection and sampling, and sampling and recovery. Across the different STs, the mean time between infection and sampling was estimated at around 10–20 days, but associated with a distribution with a long tail, such that in some individuals there could be a much longer interval (see S25 Fig for examples). Given that most patients leave the hospital before they recover, the distribution of recovery times followed the prior distribution (S24 and S25 Figs).

### Transmission rates by specialty

**Fig 6:**
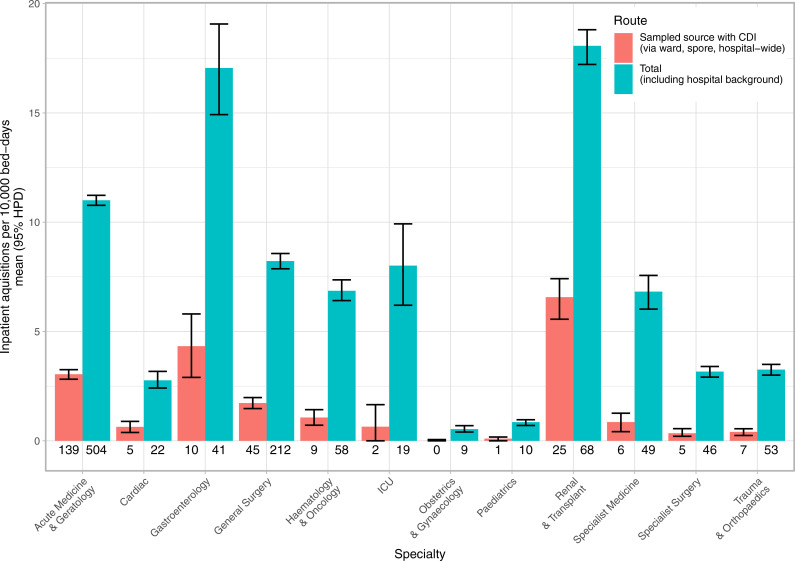
Inpatient *C*. *difficile* acquisition by specialty. The numbers at the bottom of each bar indicate the total number of CDI cases in each category.

Inferred rates of inpatient acquisition of *C*. *difficile* varied by specialty. Of 1092 infections estimated to have occurred in the hospital, 504 were acquired while patients were cared for by the acute medicine and geratology service and 212 on general surgery units. The overall rate of inpatient acquisition was 6.7 per 10,000 bed-days, with the highest rates seen on the renal and transplant, 18.1 (95%CI 17.2–18.8), gastroenterology 17.1 (14.9–19.1) and acute medicine / geratology wards 11.0 (10.8–11.2) (Fig 6). Rates of acquisition from a known case per 10,000 bed-days were also greatest in the same three specialties, 6.6 (5.6–7.4), 4.3 (2.9–5.8) and 3.0 (2.8–3.26) respectively.

### Between hospital comparisons

**Fig 7:**
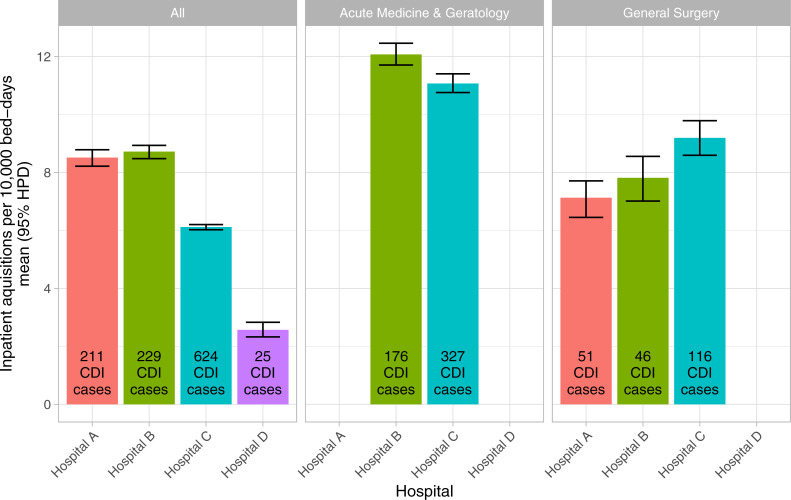
Inpatient *C*. *difficile* acquisition by hospital.

Four hospitals serve the county of Oxfordshire, hospital A (cancer centre, specialist medicine and renal services), hospital B (general district hospital), hospital C (providing acute medicine and surgery, specialist surgery, maternity and children’s services), and hospital D (elective orthopaedics and rheumatology). Overall rates of inpatient *C*. *difficile* acquisition were 6.1 (95%CI 6.0–6.2), 8.5 (8.2–8.8), 8.7 (8.5–8.9), and 2.6 (2.3–2.8) per 10000 bed-days respectively. However, these differences were largely explained by case mix given the different services based in each hospital (see Fig 5). Incidence rates in acute medicine and geratology were marginally higher at the hospital B, and rates in general surgery higher at hospital C (Fig 7).

### Temporal patterns in C. difficile acquisition

**Fig 8:**
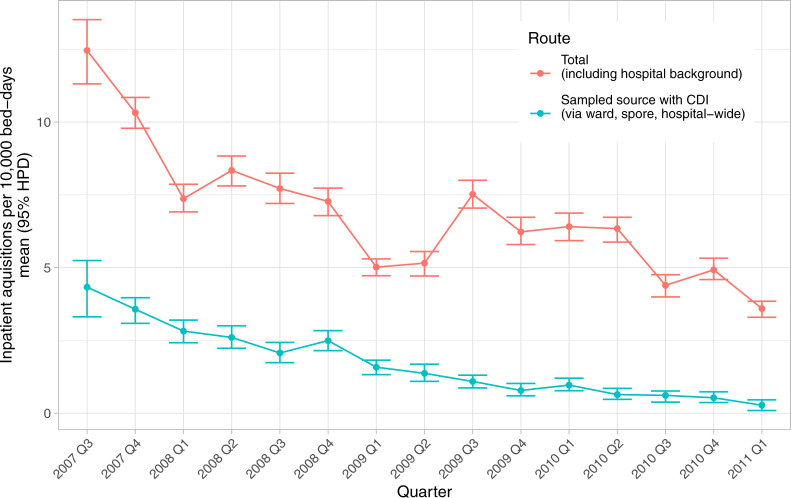
Temporal trends in *C*. *difficile* inpatient acquisition. Q, quarter; CDI, *C*. *difficile* infection.

Overall rates of inpatient *C*. *difficile* fell over the course of the study from 12.6 (95%CI 11.3–13.5) per 10000 bed-days in the third quarter of 2007 to 3.6 (3.3–3.8) in the first quarter of 2011. Perhaps even more strikingly by the end of the study period inpatient acquisition from a known case had been almost completely controlled, rates falling from 4.3 (3.3–5.2) to 0.3 (0.1–0.5) respectively (Fig 8).

## Discussion

Here we combine detailed epidemiological data on patient contact networks in hospital with whole-genome sequencing to quantitively track transmission of healthcare-associated infection. We do this in a probabilistic framework that allows principled estimates of the relative contribution of different routes of infection, differences between strains and trends over time. Using the example of *C*. *difficile* transmission, we show several important differences between key lineages.

Several methodological improvements are made over previous similar models combining epidemiological and sequencing data. We present a novel approach to using genetic data based on coalescent theory that accounts for recombination (as the input phylogenetic tree can be adjusted for recombination). We also extend the epidemiological model to include recovery, readmissions, and additional routes of transmission including via persisting ward contamination and across different hospital wards.

Through simulation we demonstrate our model performs appropriately. We also show that in this setting point estimates from a simple heuristic rule together with genomic data can outperform a sophisticated epidemiological model run without genomic data, albeit without quantification of uncertainty. Genomic data is particularly helpful here in the setting of an endemic disease with significant genomic diversity present. In an outbreak setting with limited genetic diversity it is likely that detailed epidemiological data could determine much more of the inference. However, while providing reasonable estimates overall, the errors made by the heuristic rule show substantial biases. This finding suggests earlier studies using similar rules are likely to have under-estimated the extent of transmission via ward-based contamination and hospital-wide transmission. This provides a notable shift in our understanding of transmission: we previously reported [2] that transmission via these routes accounted for 1% and 3% of cases respectively, but now estimate these to be higher 8% (95% CI 6–9%) and 4% (3–5%), with the lower bounds of the 95% credibility intervals exceeding the previous estimates.

In *C*. *difficile* it is increasingly understood that different lineages show different transmission properties and inhabit differing environmental niches, with some lineages associated with healthcare, and others with wide geographic dispersal and likely community-based acquisition [24]. Several ribotypes have been disproportionately associated with healthcare-associated disease in epidemiological surveys and reports of outbreaks [18,26], e.g. ribotypes 027, 001, 106, 176, 018/356, as well as showing evidence of geographic clustering consistent with predominantly local spread [24]. The success of these lineages in healthcare has been hypothesised to have been driven by their widespread fluroquinolone resistance and high levels of use of these antibiotics [10]. In addition to this, here we provide further insights into the healthcare-adaptation of these lineages. We show several are associated with higher rates of ward-based transmission per infected case, including ST1 (ribotype-027), ST3 (ribotype-001) and ST42 (ribotype-106). Furthermore, we show spores from ST1 persisted longer than those from all other STs other than ST3, ST42 and the other group (S20 and S25 Figs). This in turn was reflected in ST1, ST3 and ST42 having the greatest proportion of cases acquired from spores, 31%, 11% and 19% respectively (Fig 4E). It is likely that the success of these lineages is multifactorial and enhanced environmental persistence may be part of this. However, it is important to acknowledge that as well as increased acquisition, increased susceptibility to infection with specific lineages, and therefore subsequent detection, could also underlie some of the ST-specific differences seen. These insights are possible because of the Bayesian approach taken, which provides the parameter estimates that previous heuristic approaches do not. We estimate that spores from ST1 may have persisted, despite hospital cleaning, for up to 100 days. This is plausible given previous descriptions of *C*. *difficile* spores persisting on undisturbed environmental surfaces for up to 5 months [27] and in isolation side rooms for ≥4 weeks [28].

We were able to estimate transmission parameters for all ST groups, as evidenced by the differences between the posterior and prior estimates (S15–S19 Figs). However, for most STs other than ST1, ST3 and ST42, spore-based transmission was too infrequent in the dataset to allow spore parameters to be estimated (S20, S21 and S22 Figs). At present we model environmental contamination as decaying geometrically, analogous to exponential decay in continuous time, so the temporal course of spores can be captured by a single parameter. In other settings where more data are available on environmental contamination the model could be extended to fit alternative temporal patterns for contamination removal, and possibly relevant covariates, such as cleaning audit data, etc.

Although we have referred to persistent ward contamination being mediated via spores left in the environment, it is also possible that transmission mediated by unsampled patients and staff also contribute to estimates of ward contamination. Where these individuals acquire *C*. *difficile* from a sampled infected patient, further onward transmission will result in cases with *C*. *difficile* genetically similar to the original sampled case. As such, these secondary cases are likely to be attributed to the index patient (and spore-based transmission if the patient has left the ward / recovered), rather than hospital background (which would typically result in a genetically distinct infection).

Our observations also support previous findings that several major *C*. *difficile* types are predominately acquired from sources other than infected patients, e.g. ST8 (ribotype-002), ST2 (ribotype-020/014), ST10 (ribotype-015), which have been previously noted to not show evidence of geographic structure and have been hypothesised to be transmitted from geographically dispersed sources such as food [24].

Our findings have implications for infection control. For example, risk-based stratification of infection control precautions could be used to target infections caused by healthcare-associated lineages, such as ribotype-027 and ribotype-001 which are more likely to spread person-to-person and contaminate the environment, e.g. with isolation, contact precautions and enhanced environmental cleaning to target more persistent spores. In contrast, strict precautions may be unnecessary for other lineages with low transmission potential. Such an approach has already been implemented in one institution without adverse outcomes [29]. The wide role for background transmission suggests a need for renewed focus on universal standard precautions to prevent acquisition from undiagnosed sources.

By using detailed epidemiological data, we are also able to show that we can estimate how the rate of transmission from other cases varies by specialty or hospital. We saw the highest rates of transmission within our renal and transplant, gastroenterology and acute medical wards. Although these areas also had the highest overall incidence, had it been known at the time that these areas also had the highest proportion of cases acquired from other cases, as opposed to other sources, this could have directed infection control efforts in a more targeted manner. It should be noted that we do not fit different parameters for each ward (as the number of ward types multiplied by the number of STs would result in very large number of parameters to estimate given the data), and so these differences are likely to be under-estimated. We also show that during the period of the study the proportion of cases acquired from another case fell, such that by the start of 2011 near complete control of transmission from other known cases was achieved. Again, had this insight been available at the time, this likely would have contributed substantially to discussion around how to further reduce incidence. Although we do not allow transmission parameters to vary by time, by fitting different models for each ST, and through these changing in incidence over time, we are able to see these effects. As we have highlighted previously the fall in incidence was largely driven by the near disappearance of ST1 from our hospitals [10] (S11 Fig). An extension of our approach would be to model the extent to which transmission parameters change over time, e.g. for ST1, although we have recently described an approach for doing this [30], we judged there not to be sufficient power to do this in the current study for all STs.

The main benefit of including genetic data in our *C*. *difficile* analysis was to allow assignment between background and the non-background sources, with epidemiological data then contributing to informing the most likely of any non-background sources. We set relatively tight priors for the *C*. *difficile* mutation rate and within-host diversity based on previous studies [2]. Our posterior estimates for these parameters were consistent with the chosen priors (S23 Fig). The population-level diversity parameter varies by the extent of within-ST diversity (S13 and S23 Figs). In specifying our model, it was not our intention necessarily to estimate all the genetic parameters, but rather that these could be used to inform transmission estimates, in particular partitioning cases into those sufficiently related to previous cases for direct transmission to be plausible and those that are likely acquired via a background route (i.e. genetically distinct). As such, where the extent of population and within-host diversity are markedly different (e.g. ST2, ST5) the precise estimates for *N*_*e*_ and *N*_*pop*_ are less important, but where there is less diversity, i.e. greater uncertainty about background vs. non-background transmission, then estimates for these parameters matter more (e.g. ST1). If our model is used for other organisms it may work best in settings where at least within-host diversity and mutation rates are already described, which is the case for many of the major bacterial pathogens.

There are several limitations to our approach. Firstly, caution should be taken when interpreting the relative attribution of cases to hospital background versus community background sources. This distinction is driven by the estimated distribution of times between disease acquisition and sampling. This is assumed to follow a negative binomial distribution and to be the same in the community and hospital. This is an over-simplification, as greater antibiotic exposure in hospital or the run-up to admission make transition from a colonised to symptomatic state more likely [31]. It is therefore possible that the time delay between acquisition and symptom onset and therefore sampling may be substantially longer in the community than hospital. Were this to be the case this would increase the number of cases attributed to community acquisition, however there are insufficient data in our current dataset to determine this. In particular, we do not have data on antibiotic prescriptions in hospital or the community, but these could be incorporated if they were available [30].

We assume that the diagnostic testing done is complete and perfectly sensitive. Under this simplifying assumption any case that is in fact acquired from an unsampled case could be attributed to the hospital background, or alternatively to spore-based transmission as discussed above. An extension to our model would be to allow for imperfectly sensitive testing and augmentation of genetic distance data for cases with false negative results. However, this would still not capture unsampled cases, which would be much more challenging given the presence of ~200–500 non-cases for every diagnosed case. We also assume that cases are equally infectious from acquisition to recovery, i.e. we do not differentiate asymptomatic and symptomatic states or the time that patients were placed into isolation, as we have no data on patient symptoms or patient locations within hospital wards (side room vs open bay). One refinement might be to allow infectiousness to vary before and after sampling, as sampling is likely to be associated with implementation of isolation and contact precautions (this was hospital policy at the time). However, the limitation regarding details on patient symptoms would remain. We also make a simplifying assumption that there is no genetic bottleneck at transmission, which may be reasonable if large numbers of organisms are acquired in faeco-oral transmission, but this assumption is untested.

In conclusion, our method provides a novel approach for integrating pathogen genomic data into stochastic individual patient transmission models. We demonstrate it can be applied at scale to study transmission of an endemic disease. It has potential to be applied across a range of pathogens. Using the methods developed for modelling the transmission of *C*. *difficile* we gain new insights and show that healthcare-associated lineages, such as ST1/ribotype-027, both transmit more readily and persist for longer in the environment, which has important implications for their effective control.

## Supporting information

S1 Fig MCMC algorithm outline. (PDF)

S2 Fig Transmission route inference, performance on simulated data, scenario: hospital background & ward transmission only. Data for 10 simulations are shown, with the number of infections attributed to a specific source type plotted in its own column and colour. Crosses indicate the simulated value, circles the estimated value (posterior mean) and the error bars the 95% highest posterior density interval.
(PDF)

S3 Fig Transmission route inference, performance on simulated data, scenario: hospital background, ward and hospital-wide transmission. Data for 10 simulations are shown, with the number of infections attributed to a specific source type plotted in its own column and colour. Crosses indicate the simulated value, circles the estimated value (posterior mean) and the error bars the 95% highest posterior density interval.
(PDF)

S4 Fig Transmission route inference, performance on simulated data, scenario: hospital background, ward, hospital-wide and short-duration spore transmission. Data for 10 simulations are shown, with the number of infections attributed to a specific source type plotted in its own column and colour. Crosses indicate the simulated value, circles the estimated value (posterior mean) and the error bars the 95% highest posterior density interval.
(PDF)

S5 Fig Transmission route inference, performance on simulated data, scenario: hospital background, ward, hospital-wide and long-duration spore transmission. Data for 10 simulations are shown, with the number of infections attributed to a specific source type plotted in its own column and colour. Crosses indicate the simulated value, circles the estimated value (posterior mean) and the error bars the 95% highest posterior density interval.
(PDF)

S6 Fig Transmission route inference, performance on simulated data, scenario: hospital background, ward, hospital-wide, spore and community transmission. Data for 10 simulations are shown, with the number of infections attributed to a specific source type plotted in its own column and colour. Crosses indicate the simulated value, circles the estimated value (posterior mean) and the error bars the 95% highest posterior density interval.
(PDF)

S7 Fig Infection time inference, performance on simulated data. Data for 50 simulations are shown. Circles indicate an exact match between the simulated and estimated date of infection, triangles the proportion of cases where the 95% highest posterior density interval captures the true simulated value.
(PDF)

S8 Fig Parameter estimates for simulated data. Each row of panels shows a different parameter and each column and colour a different simulation scenario. The dashed lines indicate the simulated value, circles the estimated value (posterior mean) and the error bars the 95% highest posterior density interval (HPD).
(PDF)

S9 Fig Parameter estimate for simulated data: sensitivity analysis for lower transmission rates. The dashed lines indicate the simulated value, circles the estimated value (posterior mean) and the error bars the 95% highest posterior density interval (HPD).
(PDF)

S10 Fig The impact of simulation population size on model precision, calibration and power. The same underlying transmission scenario–allowing for transmission by hospital background, ward-based transmission, hospital-wide transmission and spores–is simulated at different population sizes shown in each column. As the population size decreases (from left to right) uncertainty in parameter estimates increases, but the model remains well calibrated, i.e. the 95% highest posterior density (HPD) captures the simulated value, shown as a dashed line.
(PDF)

S11 Fig Oxfordshire *C*. *difficile* MLST sequence types, overall incidence and incidence per quarter 2007–2011. (PDF)

S12 Fig Maximum likelihood phylogeny of 1223 Oxfordshire *C*. *difficile* genomes, 2007–2011. The ten most common sequence types are shown as different coloured tips. Drawn using RaxML version 8.2.10 with a GTR substitution model and a gamma model of rate heterogeneity.
(PDF)

S13 Fig Distribution of pairwise single nucleotide polymorphism (SNP) differences within Oxfordshire *C*. *difficile* sequence types (STs). For visualisation purposes differences between 3 ST3 genomes substantially differing by >5000 SNPs from all other sequenced ST3 genomes are omitted.
(PDF)

S14 Fig Example MCMC traces, from 3 chains run for ST2. Traces are shown for the six transmission parameters and the overall posterior value. Final parameter estimates were obtained by merging the output of each of the chains. The burn in period is not shown, as starting values were sufficiently different to the posterior estimates to make visualisation of mixing uninformative. The effective sample size (ESS) is shown for each parameter.
(PDF)

S15 Fig Oxfordshire *C*. *difficile* transmission rate parameters by sequence type (ST). Prior not plotted, but for all 4 parameters, mean 3.4e-03 (95% HPD 0.5e-03–11.1e-03). Note that background parameters depend on the relative prevalence of each ST, hence higher values for “Other” STs, which is the most prevalent group overall (see S11 Fig).
(PDF)

S16 Fig Oxfordshire *C*. *difficile* transmission rate parameters by sequence type (ST): hospital background. Note that background parameters depend on the relative prevalence of each ST, hence higher values for “Other” STs, which is the most prevalent group overall (see S11 Fig).
(PDF)

S17 Fig Oxfordshire *C*. *difficile* transmission rate parameters by sequence type (ST): ward-based transmission. (PDF)

S18 Fig Oxfordshire *C*. *difficile* transmission rate parameters by sequence type (ST): between ward, hospital-wide, transmission. (PDF)

S19 Fig Oxfordshire *C*. *difficile* transmission rate parameters by sequence type (ST): community background. Note that background parameters depend on the relative prevalence of each ST, hence higher values for “Other” STs, which is the most prevalent group overall (see S8 Fig). The prior distribution is not shown as nearly all the prior probability density is at higher values.
(PDF)

S20 Fig Oxfordshire *C*. *difficile* spore parameters, by sequence type. (PDF)

S21 Fig Oxfordshire *C*. *difficile* transmission rate parameters by sequence type (ST): spore multiplier. (PDF)

S22 Fig Oxfordshire *C*. *difficile* transmission rate parameters by sequence type (ST): spore decay probability. (PDF)

S23 Fig Oxfordshire *C*. *difficile* genetic parameter estimates, by sequence type. The prior distribution for within-host diversity, *N*_*e*,_ and mutation rate were set based on doi:10.1056/nejmoa1216064. The population-level diversity, *N*_*pop*,_ parameter reflects the diversity present within the ST, with smaller values representing less diversity.
(PDF)

S24 Fig Oxfordshire *C*. *difficile* sampling delay and recovery parameter estimates. (PDF)

S25 Fig Oxfordshire *C*. *difficile* prior and posterior (for ST1 and ST2) distributions for sampling and recovery intervals and spore duration. (PDF)

S1 Table Mean number of infections simulated by each route in simulations of the impact population size on model precision, calibration and power. Parameter estimates for these simulations are shown in S10 Fig.
(PDF)
