# Supplementary figures and images for: Probabilistic transmission models incorporating sequencing data for healthcare-associated Clostridioides difficile outperform heuristic rules and identify strain-specific differences in transmission
Source: PLoS Comput Biol. 2021 Jan 14;17(1):e1008417. doi: 10.1371/journal.pcbi.1008417 (PMC7840057; doi:10.1371/journal.pcbi.1008417)

3 steps at each iteration

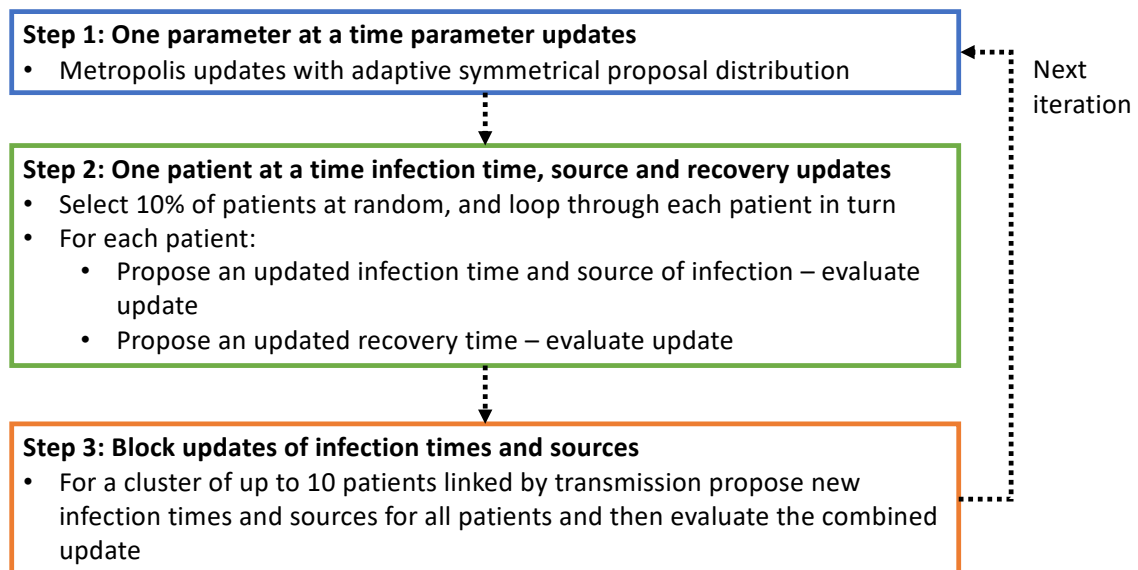

S1 Fig. MCMC algorithm outline.

Supplement: S1 Fig — (PDF) [file pcbi.1008417.s001.pdf]

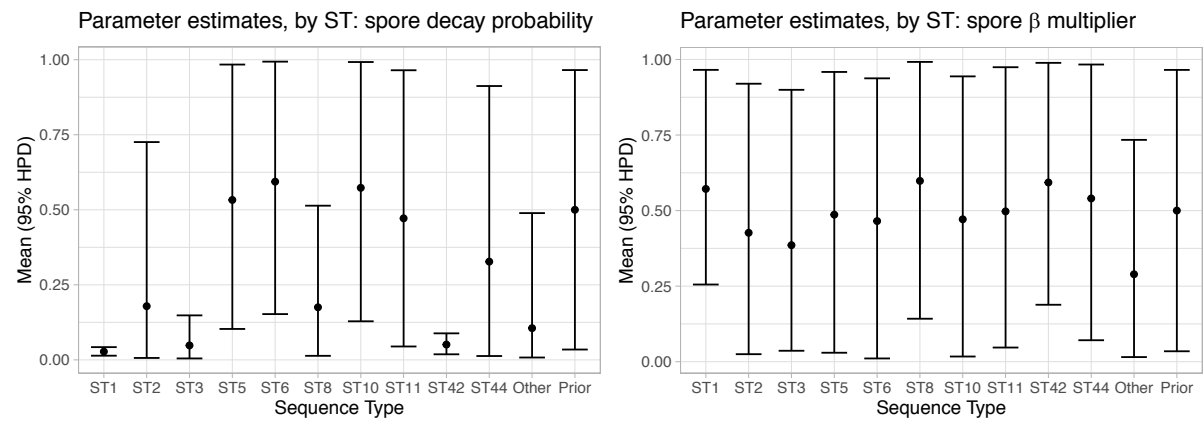

**S20 Fig. Oxfordshire *C. difficile* spore parameters, by sequence type.**

Supplement: S20 Fig — (PDF) [file pcbi.1008417.s020.pdf]

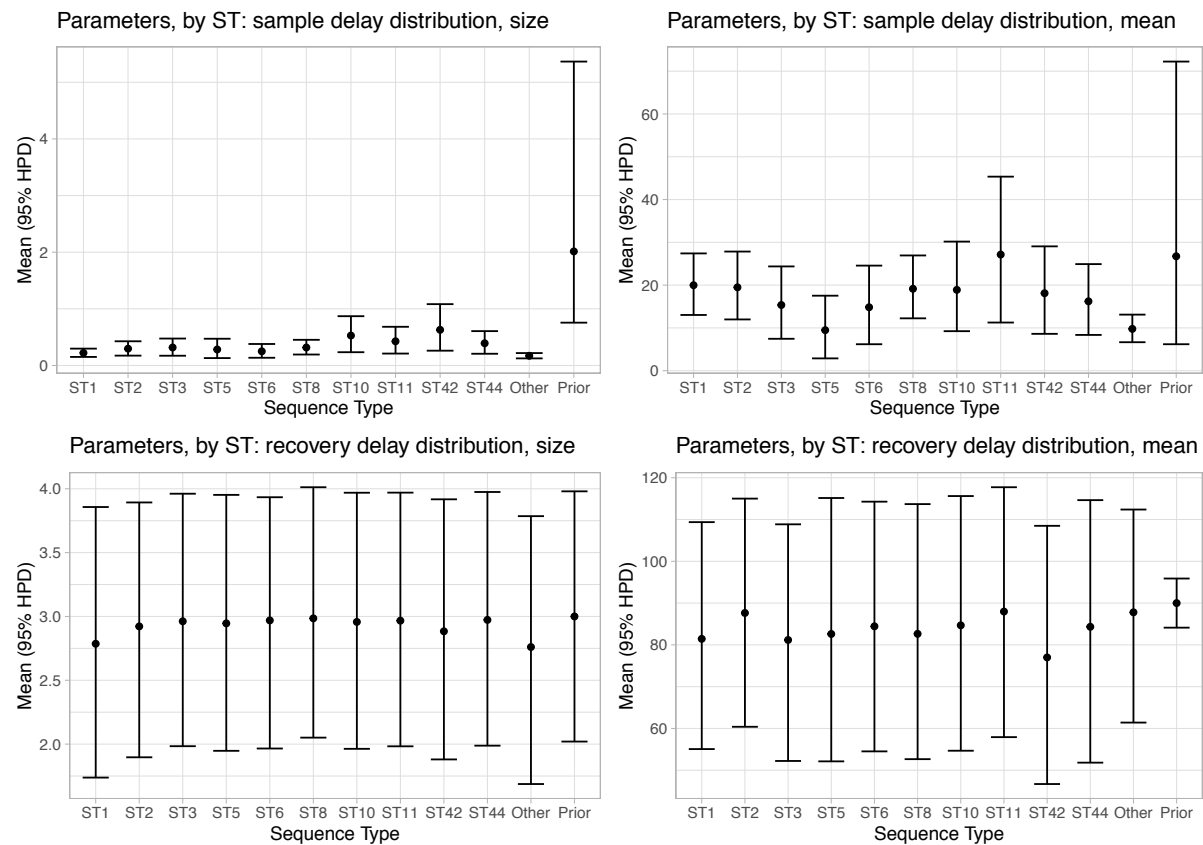

**S24 Fig. Oxfordshire *C. difficile* sampling delay and recovery parameter estimates.**

Supplement: S24 Fig — (PDF) [file pcbi.1008417.s024.pdf]
