# Supplementary material for: Probabilistic transmission models incorporating sequencing data for healthcare-associated Clostridioides difficile outperform heuristic rules and identify strain-specific differences in transmission
Source: PLoS Comput Biol. 2021 Jan 14;17(1):e1008417. doi: 10.1371/journal.pcbi.1008417 (PMC7840057; doi:10.1371/journal.pcbi.1008417)
Supplement: S4 Fig — Data for 10 simulations are shown, with the number of infections attributed to a specific source type plotted in its own column and colour. Crosses indicate the simulated value, circles the estimated value (posterior mean) and the error bars the 95% highest posterior density interval. (PDF) [file pcbi.1008417.s004.pdf]

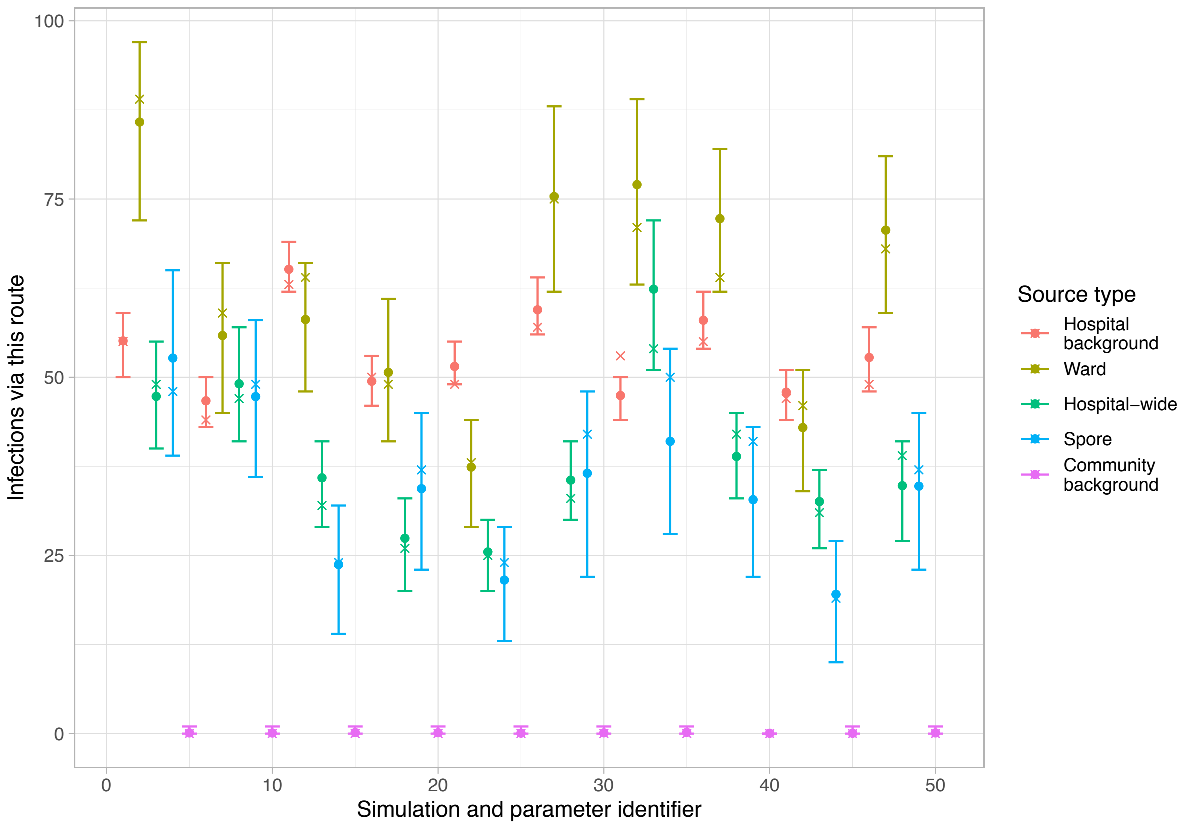

S4 Fig. Transmission route inference, performance on simulated data, scenario: hospital background, ward, hospital-wide and short-duration spore transmission. Data for 10 simulations are shown, with the number of infections attributed to a specific source type plotted in its own column and colour. Crosses indicate the simulated value, circles the estimated value (posterior mean) and the error bars the 95% highest posterior density interval.
