# Supplementary material for: Probabilistic transmission models incorporating sequencing data for healthcare-associated Clostridioides difficile outperform heuristic rules and identify strain-specific differences in transmission
Source: PLoS Comput Biol. 2021 Jan 14;17(1):e1008417. doi: 10.1371/journal.pcbi.1008417 (PMC7840057; doi:10.1371/journal.pcbi.1008417)
Supplement: S7 Fig — Data for 50 simulations are shown. Circles indicate an exact match between the simulated and estimated date of infection, triangles the proportion of cases where the 95% highest posterior density interval captures the true simulated value. (PDF) [file pcbi.1008417.s007.pdf]

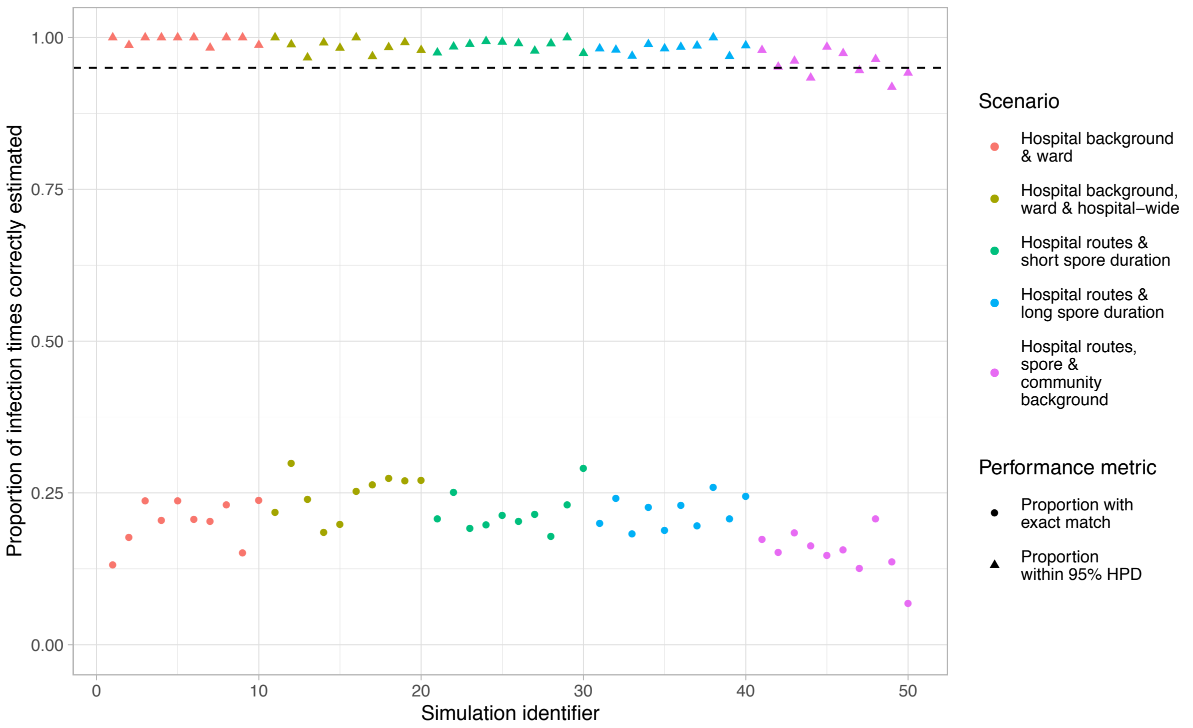


**S7 Fig. Infection time inference, performance on simulated data**. Data for 50 simulations are shown. Circles indicate an exact match between the simulated and estimated date of infection, triangles the proportion of cases where the 95% highest posterior density interval captures the true simulated value.
