# Supplementary material for: Probabilistic transmission models incorporating sequencing data for healthcare-associated Clostridioides difficile outperform heuristic rules and identify strain-specific differences in transmission
Source: PLoS Comput Biol. 2021 Jan 14;17(1):e1008417. doi: 10.1371/journal.pcbi.1008417 (PMC7840057; doi:10.1371/journal.pcbi.1008417)
Supplement: S8 Fig — Each row of panels shows a different parameter and each column and colour a different simulation scenario. The dashed lines indicate the simulated value, circles the estimated value (posterior mean) and the error bars the 95% highest posterior density interval (HPD). (PDF) [file pcbi.1008417.s008.pdf]

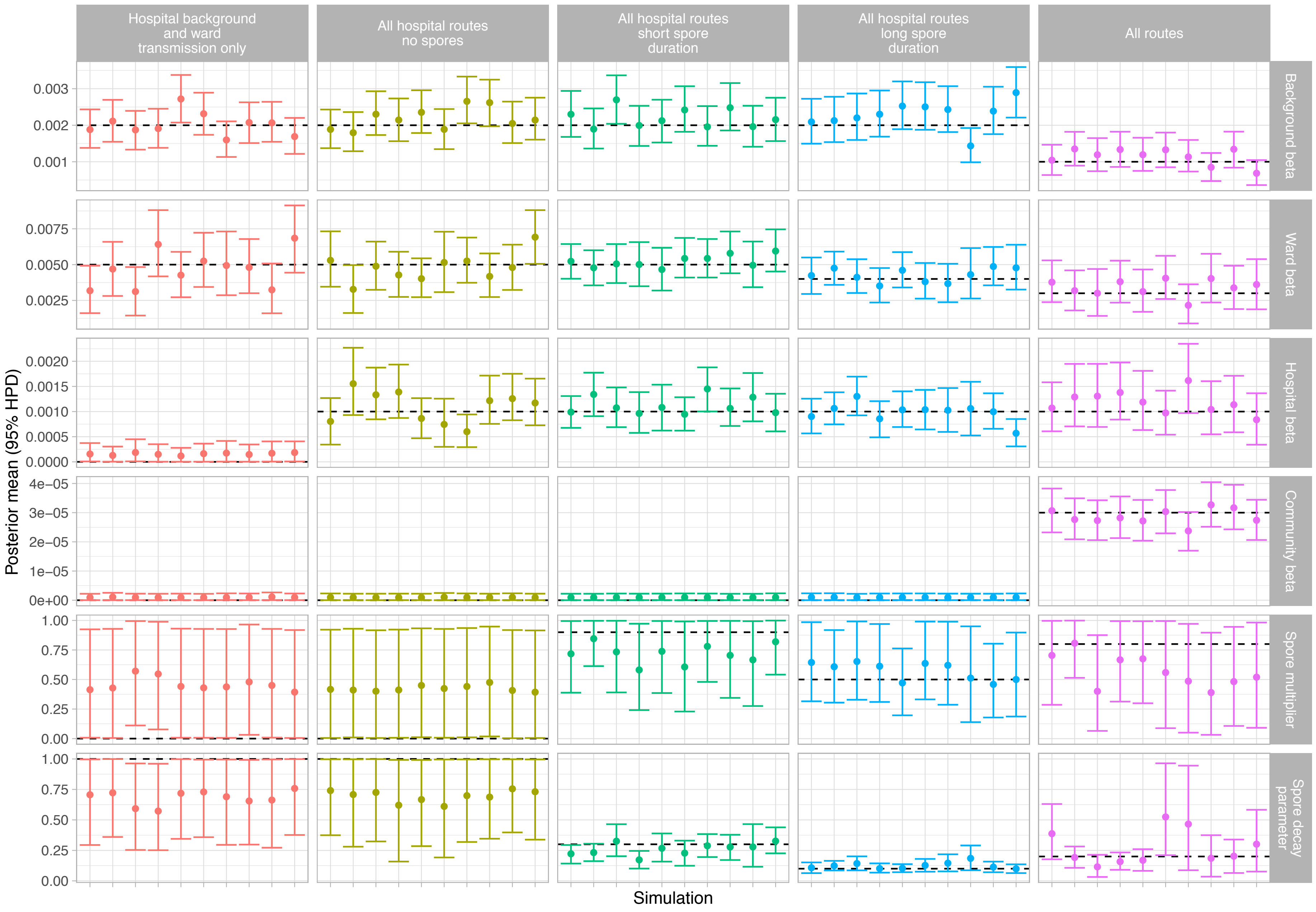

S8 Fig. Parameter estimates for simulated data. Each row of panels shows a different parameter and each column and colour a different simulation scenario. The dashed lines indicate the simulated value, circles the estimated value (posterior mean) and the error bars the 95% highest posterior density interval (HPD).
