# Supplementary material for: Probabilistic transmission models incorporating sequencing data for healthcare-associated Clostridioides difficile outperform heuristic rules and identify strain-specific differences in transmission
Source: PLoS Comput Biol. 2021 Jan 14;17(1):e1008417. doi: 10.1371/journal.pcbi.1008417 (PMC7840057; doi:10.1371/journal.pcbi.1008417)
Supplement: S9 Fig — The dashed lines indicate the simulated value, circles the estimated value (posterior mean) and the error bars the 95% highest posterior density interval (HPD). (PDF) [file pcbi.1008417.s009.pdf]

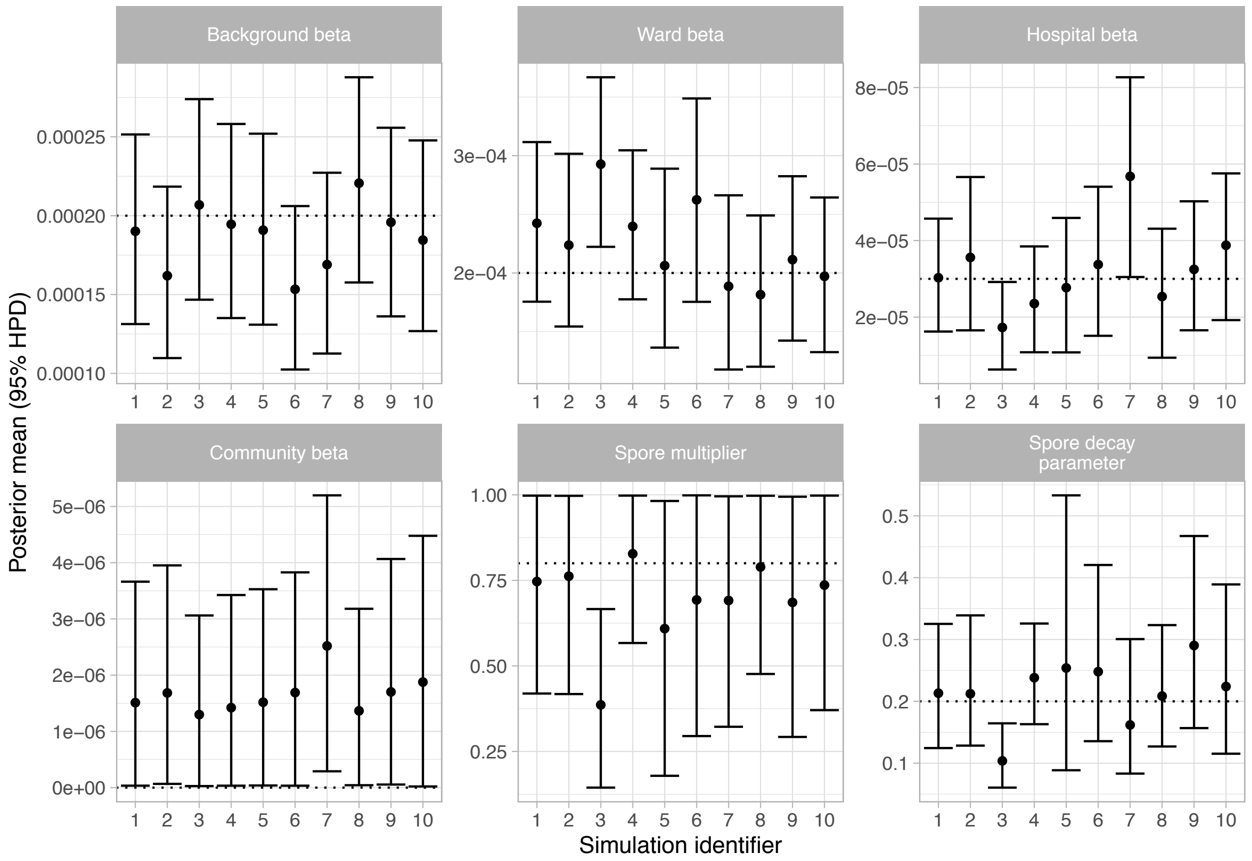

S9 Fig. Parameter estimate for simulated data: sensitivity analysis for lower transmission rates. The dashed lines indicate the simulated value, circles the estimated value (posterior mean) and the error bars the 95% highest posterior density interval (HPD).
