# Supplementary material for: Probabilistic transmission models incorporating sequencing data for healthcare-associated Clostridioides difficile outperform heuristic rules and identify strain-specific differences in transmission
Source: PLoS Comput Biol. 2021 Jan 14;17(1):e1008417. doi: 10.1371/journal.pcbi.1008417 (PMC7840057; doi:10.1371/journal.pcbi.1008417)
Supplement: S10 Fig — The same underlying transmission scenario–allowing for transmission by hospital background, ward-based transmission, hospital-wide transmission and spores–is simulated at different population sizes shown in each column. As the population size decreases (from left to right) uncertainty in parameter estimates increases, but the model remains well calibrated, i.e. the 95% highest posterior density (HPD) captures the simulated value, shown as a dashed line. (PDF) [file pcbi.1008417.s010.pdf]

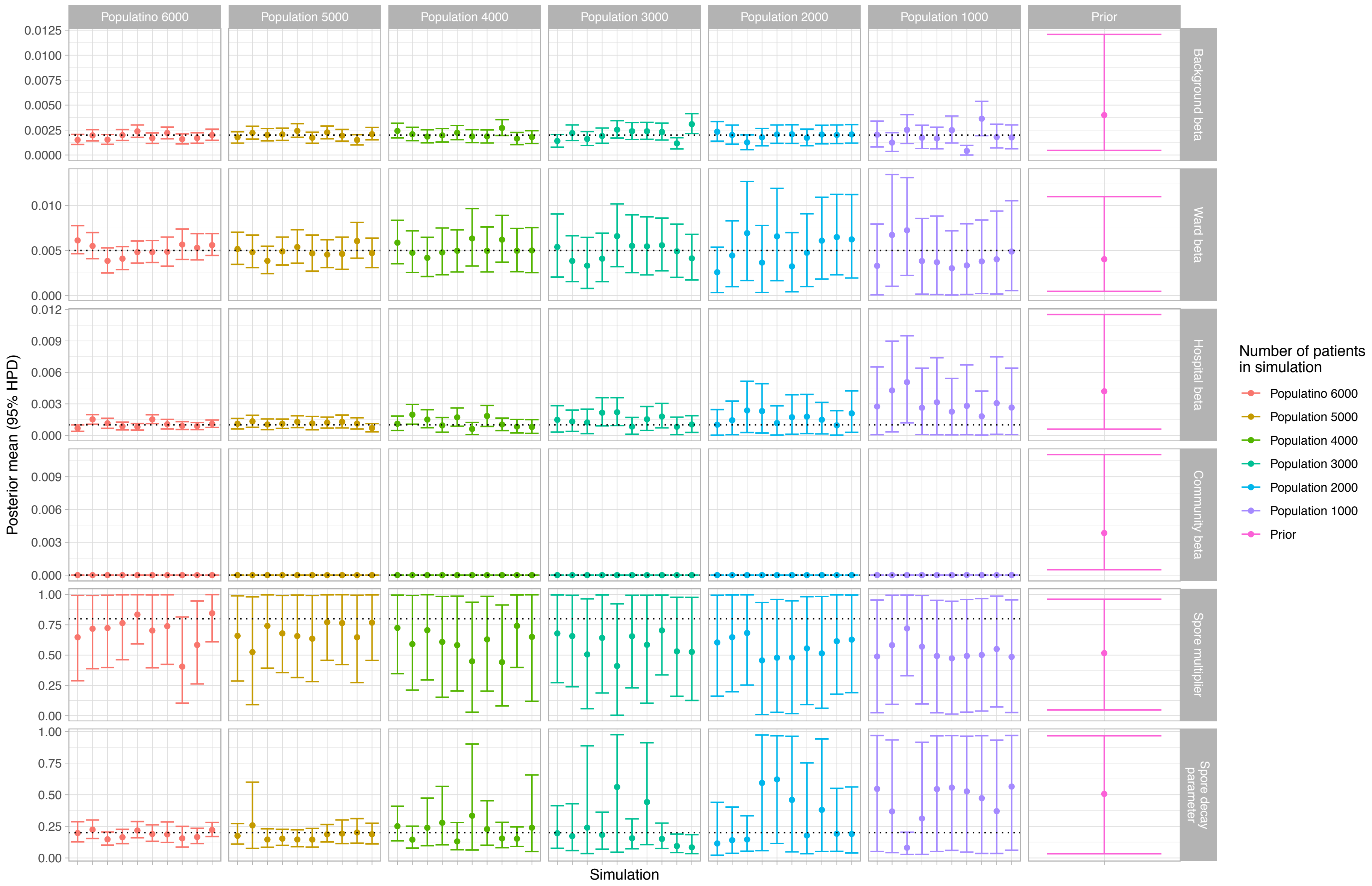

S10 Fig. The impact of simulation population size on model precision, calibration and power. The same underlying transmission scenario – allowing for transmission by hospital background, ward-based transmission, hospital-wide transmission and spores – is simulated at different population sizes shown in each column. As the population size decreases (from left to right) uncertainty in parameter estimates increases, but the model remains well calibrated, i.e. the 95% highest posterior density (HPD) captures the simulated value, shown as a dashed line.
