# Supplementary material for: Probabilistic transmission models incorporating sequencing data for healthcare-associated Clostridioides difficile outperform heuristic rules and identify strain-specific differences in transmission
Source: PLoS Comput Biol. 2021 Jan 14;17(1):e1008417. doi: 10.1371/journal.pcbi.1008417 (PMC7840057; doi:10.1371/journal.pcbi.1008417)
Supplement: S12 Fig — The ten most common sequence types are shown as different coloured tips. Drawn using RaxML version 8.2.10 with a GTR substitution model and a gamma model of rate heterogeneity. (PDF) [file pcbi.1008417.s012.pdf]

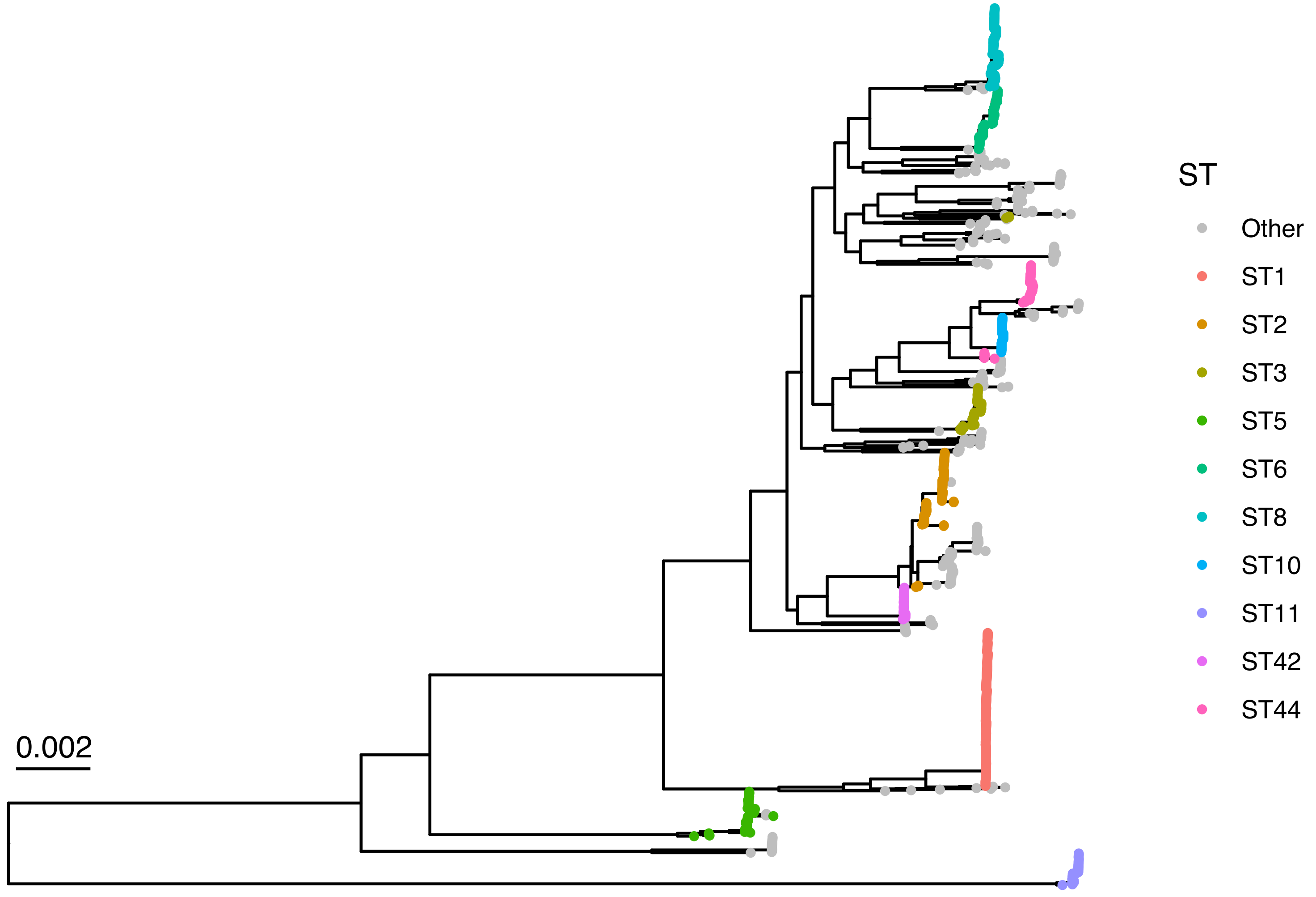

S12 Fig. Maximum likelihood phylogeny of 1223 Oxfordshire *C. difficile* genomes, 2007-2011. The ten most common sequence types are shown as different coloured tips. Drawn using RaxML version 8.2.10 with a GTR substitution model and a gamma model of rate heterogeneity.
