# Supplementary material for: Probabilistic transmission models incorporating sequencing data for healthcare-associated Clostridioides difficile outperform heuristic rules and identify strain-specific differences in transmission
Source: PLoS Comput Biol. 2021 Jan 14;17(1):e1008417. doi: 10.1371/journal.pcbi.1008417 (PMC7840057; doi:10.1371/journal.pcbi.1008417)
Supplement: S13 Fig — For visualisation purposes differences between 3 ST3 genomes substantially differing by >5000 SNPs from all other sequenced ST3 genomes are omitted. (PDF) [file pcbi.1008417.s013.pdf]

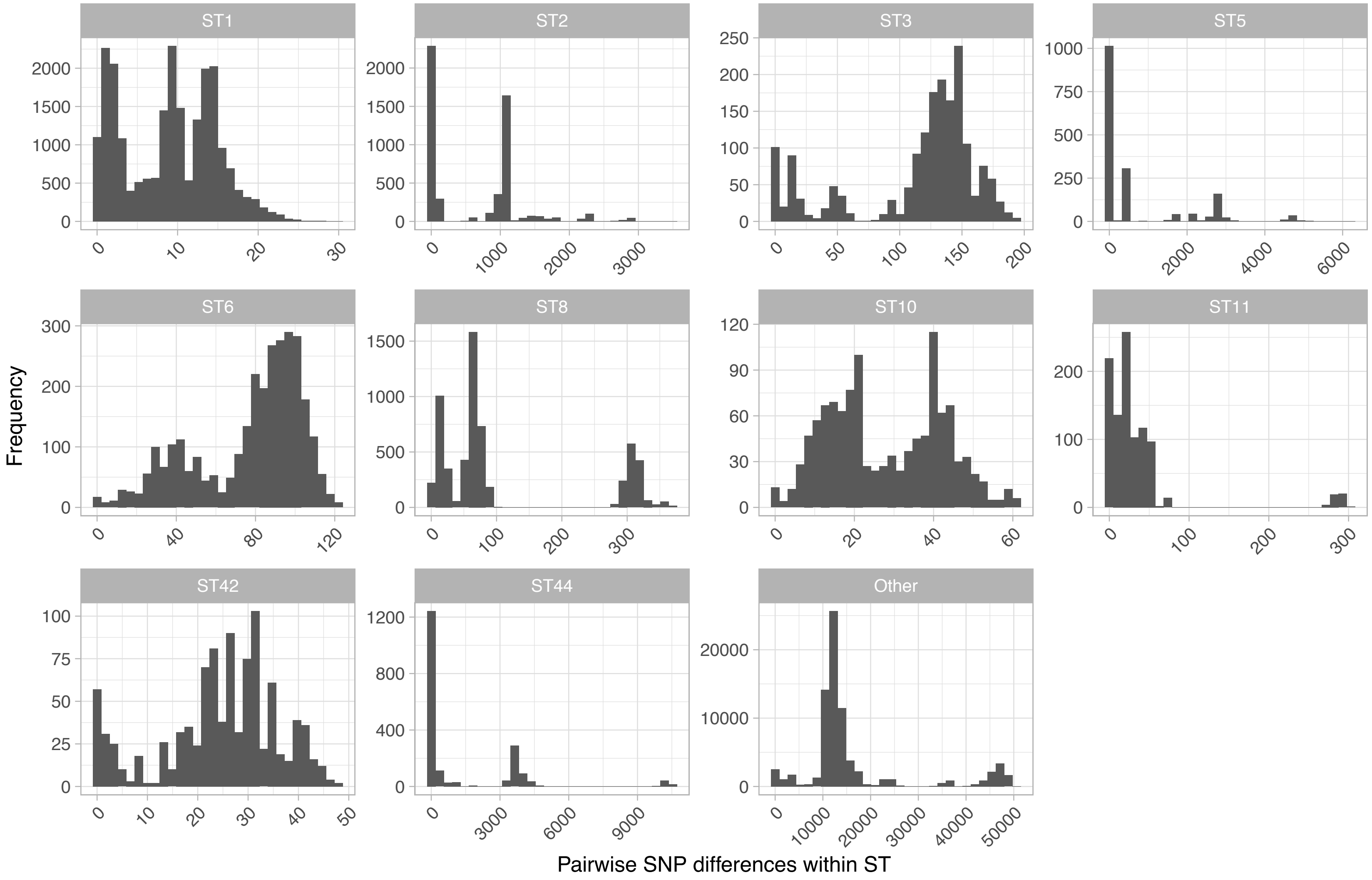

S13 Fig. Distribution of pairwise single nucleotide polymorphism (SNP) differences within Oxfordshire *C. difficile* sequence types (STs). For visualisation purposes differences between 3 ST3 genomes substantially differing by >5000 SNPs from all other sequenced ST3 genomes are omitted.
