# Supplementary material for: Probabilistic transmission models incorporating sequencing data for healthcare-associated Clostridioides difficile outperform heuristic rules and identify strain-specific differences in transmission
Source: PLoS Comput Biol. 2021 Jan 14;17(1):e1008417. doi: 10.1371/journal.pcbi.1008417 (PMC7840057; doi:10.1371/journal.pcbi.1008417)
Supplement: S14 Fig — Traces are shown for the six transmission parameters and the overall posterior value. Final parameter estimates were obtained by merging the output of each of the chains. The burn in period is not shown, as starting values were sufficiently different to the posterior estimates to make visualisation of mixing uninformative. The effective sample size (ESS) is shown for each parameter. (PDF) [file pcbi.1008417.s014.pdf]

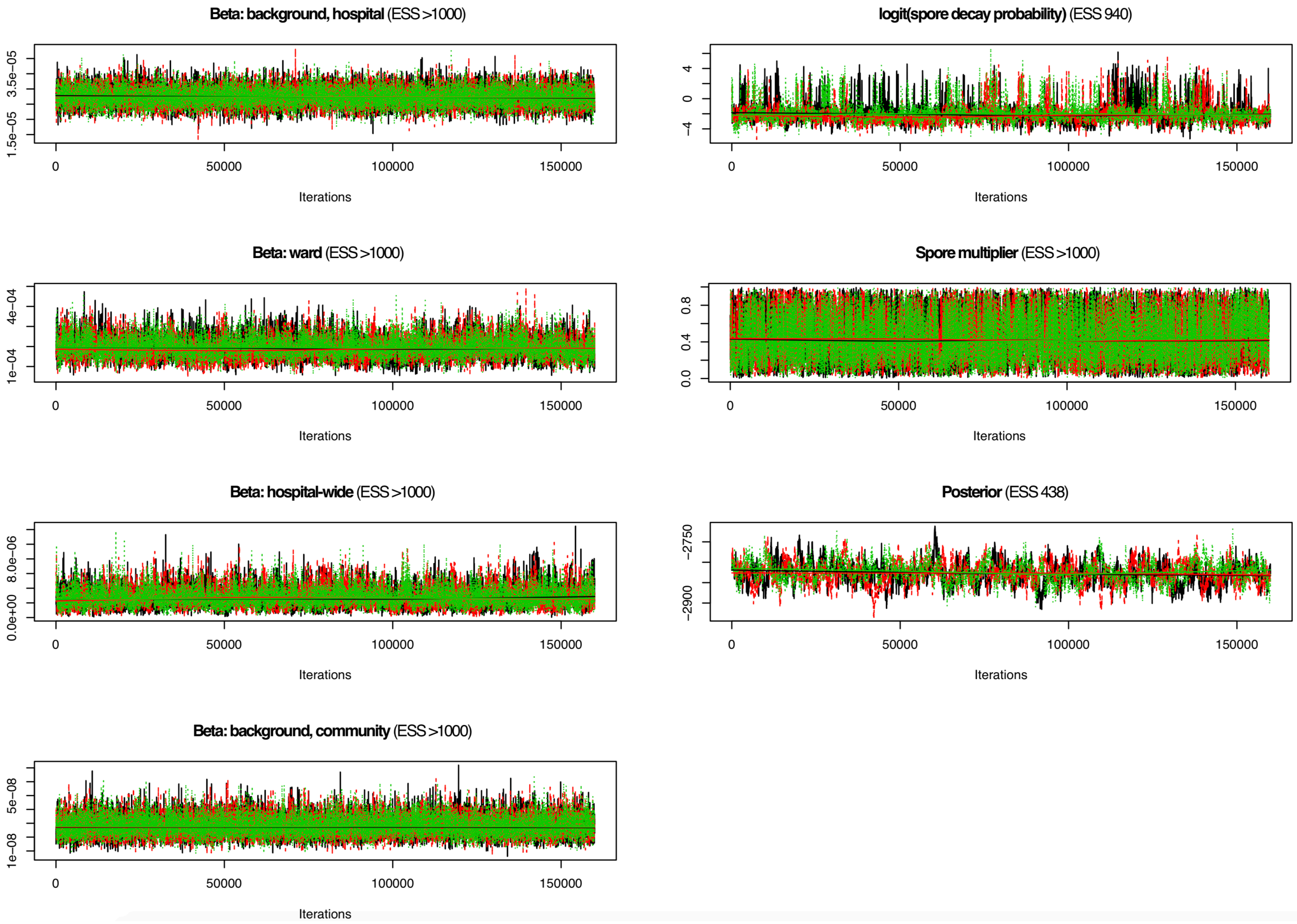

S14 Fig. Example MCMC traces, from 3 chains run for ST2. Traces are shown for the six transmission parameters and the overall posterior value. Final parameter estimates were obtained by merging the output of each of the chains. The burn in period is not shown, as starting values were sufficiently different to the posterior estimates to make visualisation of mixing uninformative. The effective sample size (ESS) is shown for each parameter.
