# Supplementary material for: Probabilistic transmission models incorporating sequencing data for healthcare-associated Clostridioides difficile outperform heuristic rules and identify strain-specific differences in transmission
Source: PLoS Comput Biol. 2021 Jan 14;17(1):e1008417. doi: 10.1371/journal.pcbi.1008417 (PMC7840057; doi:10.1371/journal.pcbi.1008417)
Supplement: S15 Fig — Prior not plotted, but for all 4 parameters, mean 3.4e-03 (95% HPD 0.5e-03–11.1e-03). Note that background parameters depend on the relative prevalence of each ST, hence higher values for “Other” STs, which is the most prevalent group overall (see S11 Fig). (PDF) [file pcbi.1008417.s015.pdf]

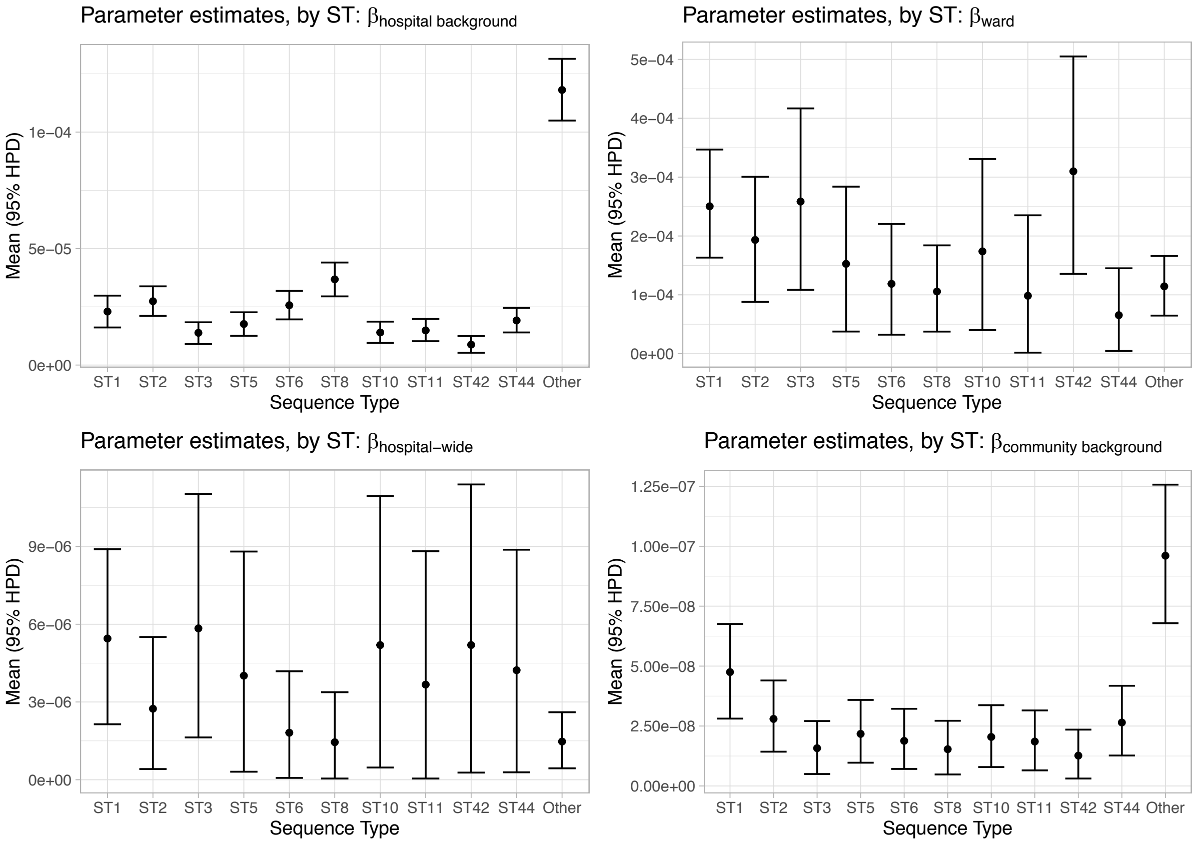

S15 Fig. Oxfordshire *C. difficile* transmission rate parameters by sequence type (ST). Prior not plotted, but for all 4 parameters, mean 3.4e-03 (95% HPD 0.5e-03 – 11.1e-03). Note that background parameters depend on the relative prevalence of each ST, hence higher values for "Other" STs, which is the most prevalent group overall (see Figure S11).
