# Supplementary material for: Probabilistic transmission models incorporating sequencing data for healthcare-associated Clostridioides difficile outperform heuristic rules and identify strain-specific differences in transmission
Source: PLoS Comput Biol. 2021 Jan 14;17(1):e1008417. doi: 10.1371/journal.pcbi.1008417 (PMC7840057; doi:10.1371/journal.pcbi.1008417)
Supplement: S16 Fig — Note that background parameters depend on the relative prevalence of each ST, hence higher values for “Other” STs, which is the most prevalent group overall (see S11 Fig). (PDF) [file pcbi.1008417.s016.pdf]

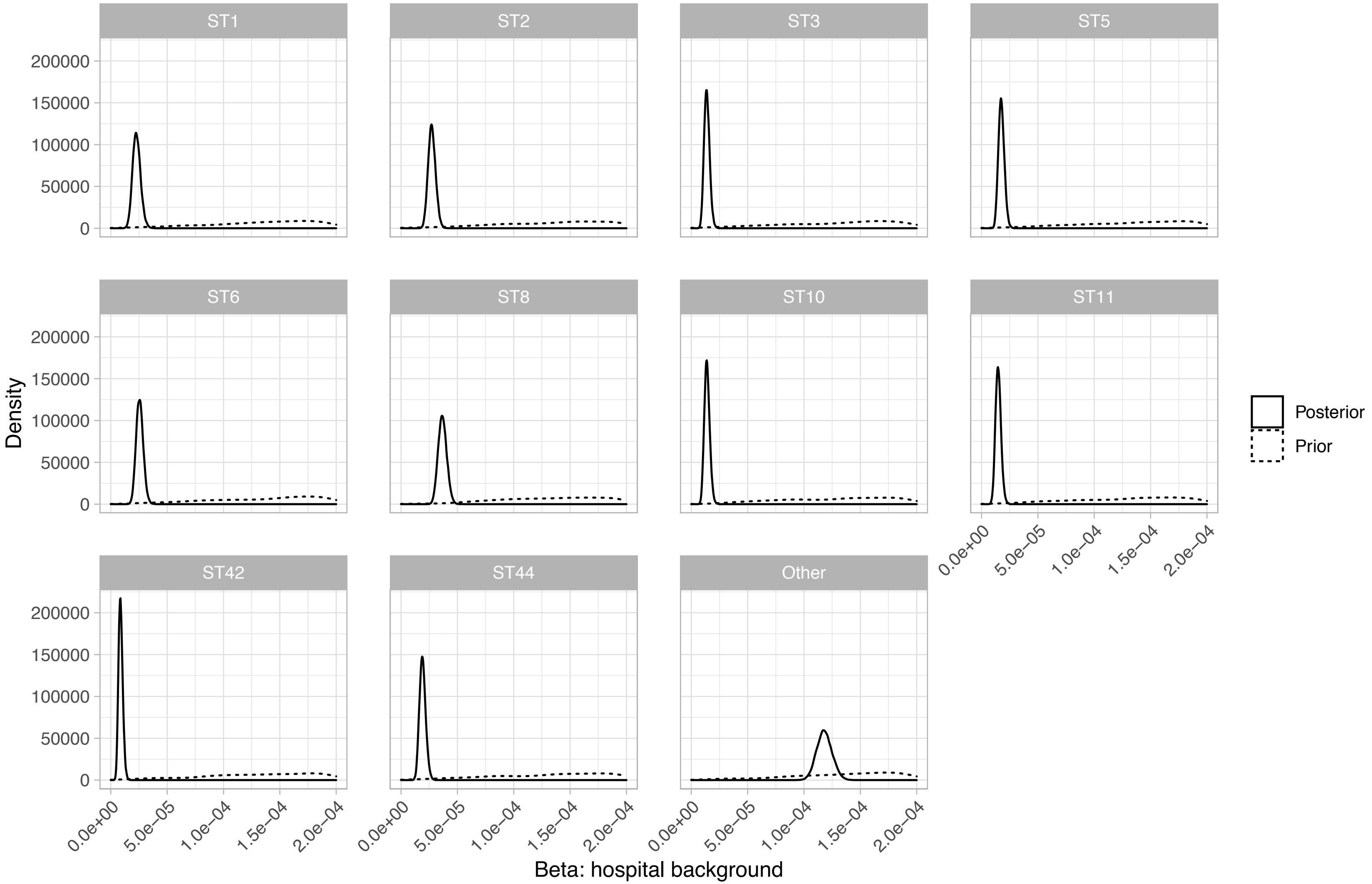

S16 Fig. Oxfordshire *C. difficile* transmission rate parameters by sequence type (ST): hospital background. Note that background parameters depend on the relative prevalence of each ST, hence higher values for "Other" STs, which is the most prevalent group overall (see Figure S11).
