# Supplementary material for: Probabilistic transmission models incorporating sequencing data for healthcare-associated Clostridioides difficile outperform heuristic rules and identify strain-specific differences in transmission
Source: PLoS Comput Biol. 2021 Jan 14;17(1):e1008417. doi: 10.1371/journal.pcbi.1008417 (PMC7840057; doi:10.1371/journal.pcbi.1008417)
Supplement: S19 Fig — Note that background parameters depend on the relative prevalence of each ST, hence higher values for “Other” STs, which is the most prevalent group overall (see S8 Fig). The prior distribution is not shown as nearly all the prior probability density is at higher values. (PDF) [file pcbi.1008417.s019.pdf]

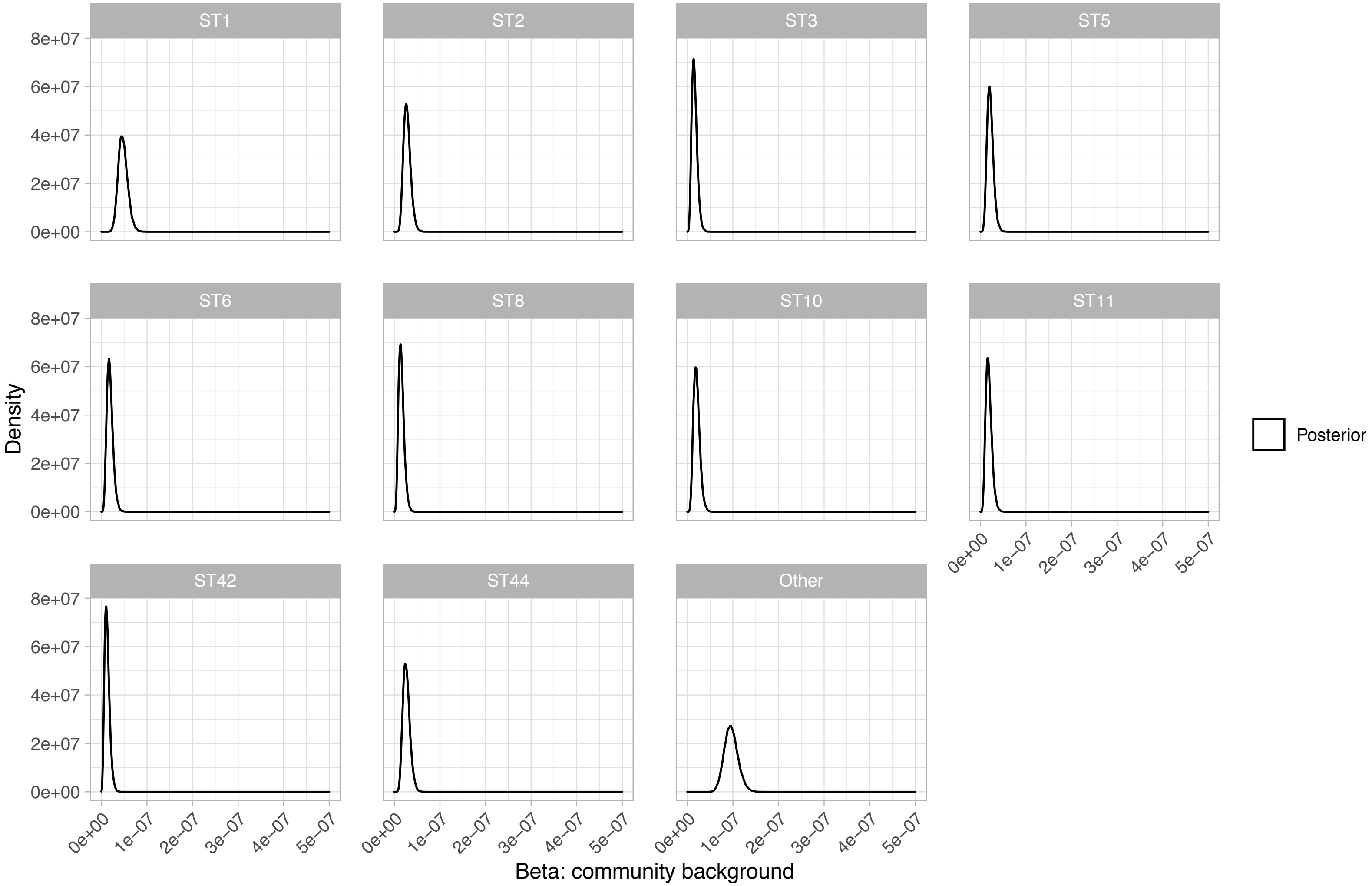

S19 Fig. Oxfordshire *C. difficile* transmission rate parameters by sequence type (ST): community background. Note that background parameters depend on the relative prevalence of each ST, hence higher values for “Other” STs, which is the most prevalent group overall (see Figure S8). The prior distribution is not shown as nearly all the prior probability density is at higher values.
