# Supplementary material for: Probabilistic transmission models incorporating sequencing data for healthcare-associated Clostridioides difficile outperform heuristic rules and identify strain-specific differences in transmission
Source: PLoS Comput Biol. 2021 Jan 14;17(1):e1008417. doi: 10.1371/journal.pcbi.1008417 (PMC7840057; doi:10.1371/journal.pcbi.1008417)
Supplement: S22 Fig — (PDF) [file pcbi.1008417.s022.pdf]

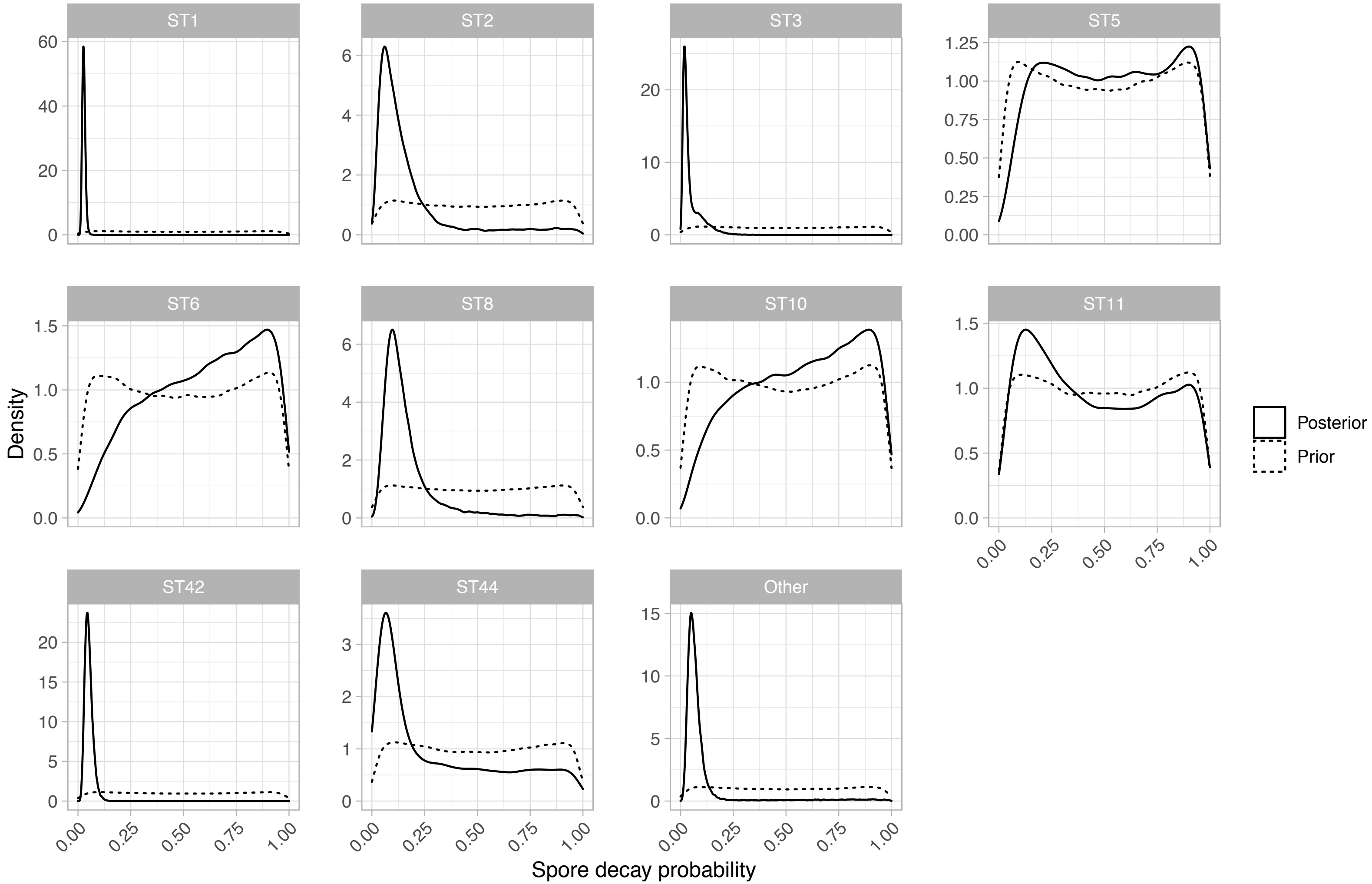

S22 Fig. Oxfordshire *C. difficile* transmission rate parameters by sequence type (ST): spore decay probability.
