# Supplementary material for: Probabilistic transmission models incorporating sequencing data for healthcare-associated Clostridioides difficile outperform heuristic rules and identify strain-specific differences in transmission
Source: PLoS Comput Biol. 2021 Jan 14;17(1):e1008417. doi: 10.1371/journal.pcbi.1008417 (PMC7840057; doi:10.1371/journal.pcbi.1008417)
Supplement: S23 Fig — The prior distribution for within-host diversity, Ne, and mutation rate were set based on doi:10.1056/nejmoa1216064. The population-level diversity, Npop, parameter reflects the diversity present within the ST, with smaller values representing less diversity. (PDF) [file pcbi.1008417.s023.pdf]

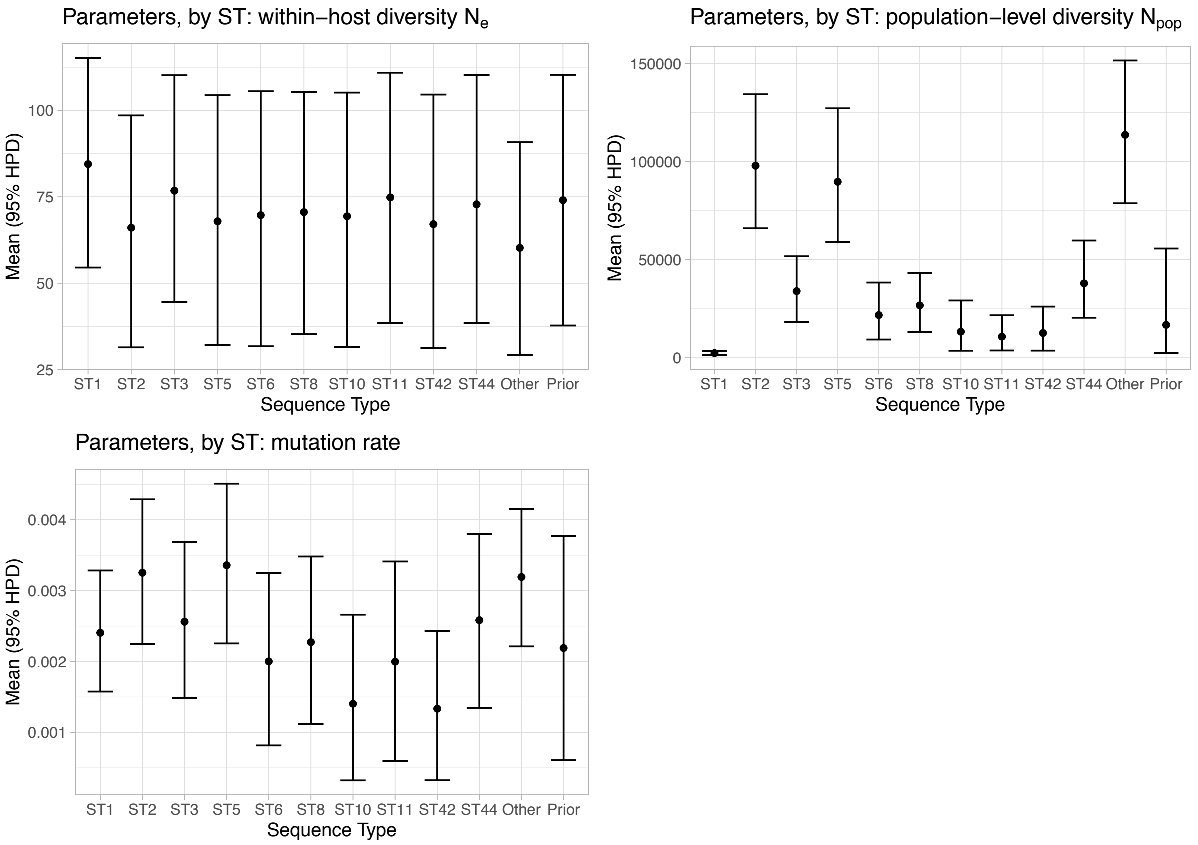

S23 Fig. Oxfordshire *C. difficile* genetic parameter estimates, by sequence type. The prior distribution for within-host diversity, N_e, and mutation rate were set based on doi:10.1056/nejmoa1216064. The population-level diversity, N_{pop}, parameter reflects the diversity present within the ST, with smaller values representing less diversity.
