# Supplementary material for: Probabilistic transmission models incorporating sequencing data for healthcare-associated Clostridioides difficile outperform heuristic rules and identify strain-specific differences in transmission
Source: PLoS Comput Biol. 2021 Jan 14;17(1):e1008417. doi: 10.1371/journal.pcbi.1008417 (PMC7840057; doi:10.1371/journal.pcbi.1008417)
Supplement: S25 Fig — (PDF) [file pcbi.1008417.s025.pdf]

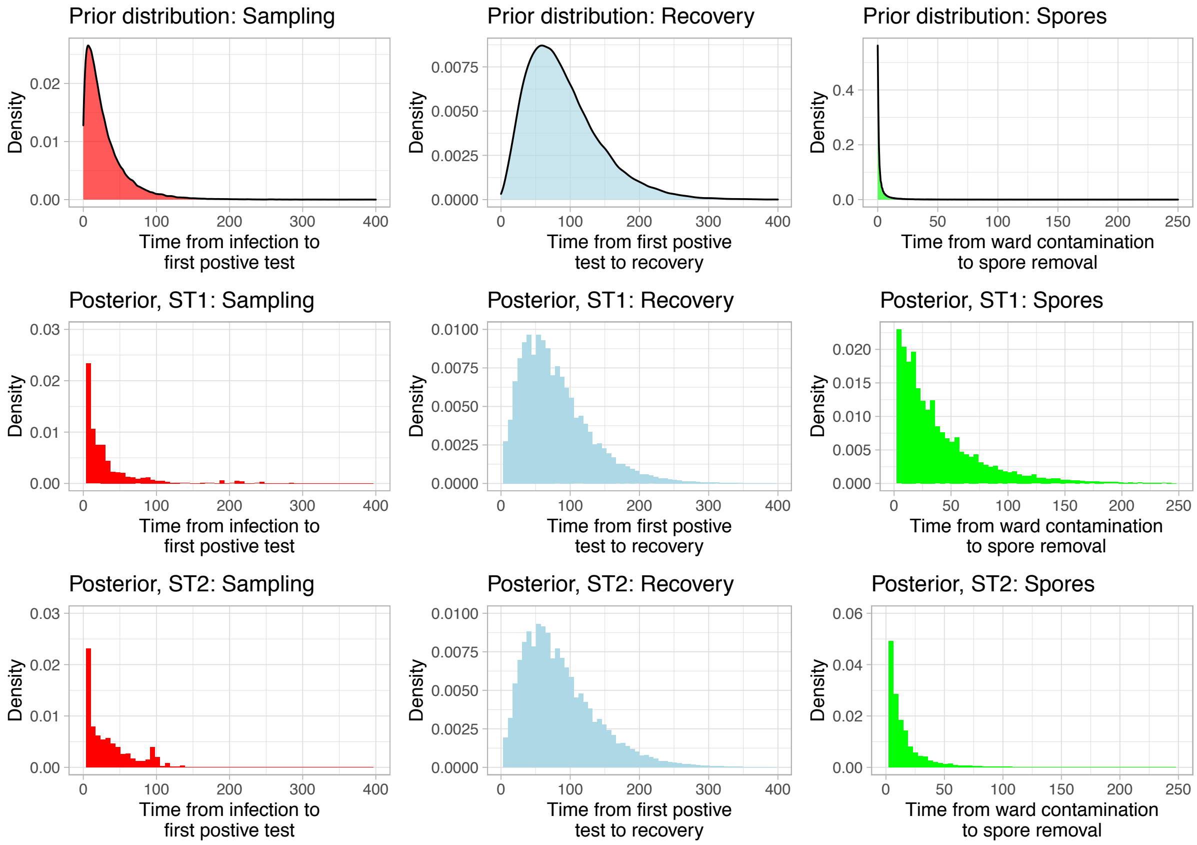

S25 Fig. Oxfordshire *C. difficile* prior and posterior (for ST1 and ST2) distributions for sampling and recovery intervals and spore duration.
