# Supplementary material for: Probabilistic transmission models incorporating sequencing data for healthcare-associated Clostridioides difficile outperform heuristic rules and identify strain-specific differences in transmission
Source: PLoS Comput Biol. 2021 Jan 14;17(1):e1008417. doi: 10.1371/journal.pcbi.1008417 (PMC7840057; doi:10.1371/journal.pcbi.1008417)
Supplement: S1 Table — Parameter estimates for these simulations are shown in S10 Fig. (PDF) [file pcbi.1008417.s026.pdf]

Population size	Mean infections via hospital background	Mean infections via hospital wards	Mean infections via across hospital transmission	Mean infections via ward-based contamination	Mean total infections
**6000**	47.5	56.0	32.2	42.6	178.3
**5000**	41.8	27.3	18.8	25.0	112.9
**4000**	33.4	14.0	9.0	10.7	67.1
**3000**	25.9	6.8	4.5	5.4	42.6
**2000**	15.5	2.1	0.6	2.2	20.4
**1000**	7.4	0.6	0.4	0.8	9.2

S1 Table. Mean number of infections simulated by each route in simulations of the impact population size on model precision, calibration and power. Parameter estimates for these simulations are shown in Figure S10.
